# Synergy of protease-binding sites within the ecotin homodimer is crucial for inhibition of MASP enzymes and for blocking lectin pathway activation

**DOI:** 10.1016/j.jbc.2022.101985

**Published:** 2022-04-25

**Authors:** Zoltán Attila Nagy, Dávid Héja, Dániel Bencze, Bence Kiss, Eszter Boros, Dávid Szakács, Krisztián Fodor, Matthias Wilmanns, Andrea Kocsis, József Dobó, Péter Gál, Veronika Harmat, Gábor Pál

**Affiliations:** 1 Department of Biochemistry, ELTE Eötvös Loránd University, Budapest, Hungary; 2 European Molecular Biology Laboratory, Hamburg Unit, Hamburg, Germany; 3 Institute of Enzymology, Research Centre for Natural Sciences, Budapest, Hungary; 4 Laboratory of Structural Chemistry and Biology, Institute of Chemistry, ELTE Eötvös Loránd University, Budapest, Hungary; 5 MTA-ELTE Protein Modelling Research Group, ELKH, Budapest, Hungary

**Keywords:** ecotin, virulence factor, X-ray crystallography, complement system, lectin pathway, mannose-binding lectin-associated serine protease, AP, alternative complement pathway, CCP, complement control protein, FD, factor D, LP, lectin pathway, MASP, mannan-binding lectin-associated serine protease, PDB, Protein Data Bank, RT, room temperature, SFMI, sunflower MASP inhibitor, SGMI, SGPI-derived MASP inhibitor, SGPI, *Schistocerca gregaria* serine protease inhibitor, SP, serine protease

## Abstract

Ecotin is a homodimeric serine protease inhibitor produced by many commensal and pathogenic microbes. It functions as a virulence factor, enabling survival of various pathogens in the blood. The ecotin dimer binds two protease molecules, and each ecotin protomer has two protease-binding sites: site1 occupies the substrate-binding groove, whereas site2 engages a distinct secondary region. Owing to the twofold rotational symmetry within the ecotin dimer, sites 1 and 2 of a protomer bind to different protease molecules within the tetrameric complex. *Escherichia coli* ecotin inhibits trypsin-like, chymotrypsin-like, and elastase-like enzymes, including pancreatic proteases, leukocyte elastase, key enzymes of blood coagulation, the contact and complement systems, and other antimicrobial cascades. Here, we show that mannan-binding lectin-associated serine protease-1 (MASP-1) and MASP-2, essential activators of the complement lectin pathway, and MASP-3, an essential alternative pathway activator, are all inhibited by ecotin. We decipher in detail how the preorganization of site1 and site2 within the ecotin dimer contributes to the inhibition of each MASP enzyme. In addition, using mutated and monomeric ecotin variants, we show that site1, site2, and dimerization contribute to inhibition in a surprisingly target-dependent manner. We present the first ecotin:MASP-1 and ecotin:MASP-2 crystal structures, which provide additional insights and permit structural interpretation of the observed functional results. Importantly, we reveal that monomerization completely disables the MASP-2-inhibitory, MASP-3-inhibitory, and lectin pathway–inhibitory capacity of ecotin. These findings provide new opportunities to combat dangerous multidrug-resistant pathogens through development of compounds capable of blocking ecotin dimer formation.

Ecotin (UniProt ID: P23827) is a reversible serine protease (SP) inhibitor first isolated from the periplasm of *Escherichia coli* (1). It represents one of the 18 independently evolved families of canonical inhibitors (2). Each family has a distinct fold but contains a surface loop responsible for the inhibition of the target enzyme. In all families, this loop adopts the same, that is, “canonical” conformation upon occupying the substrate-binding cleft of the target protease. Yet, ecotin is unique among these families as in addition to the canonical loop, which belongs to its primary binding site (site1), it also has a structurally distinct secondary binding site (site2). Moreover, ecotin is a homodimer; therefore, it has two pairs of site1 and site2. The two protomers are mainly held together through their C-terminal arms, and the homodimer has a twofold rotational symmetry. In the homodimer, site1 of one protomer and site2 of the other one are positioned such that together they can “chelate” the target proteinases (3, 4, 5). Because of the twofold symmetry, ecotin has two identical site1/site2 “chelators” and can simultaneously bind two proteinases. Explanatory Figure 1 illustrates this binding mechanism on a trypsin:ecotin complex (Protein Data Bank [PDB] ID: 1EZU; Gillmor *et al.* (5)) that allows for easier comprehension of our new mannan-binding lectin-associated serine proteinase (MASP):ecotin crystal structures shown in Figure 2 of the Results section.

**Figure 1:**
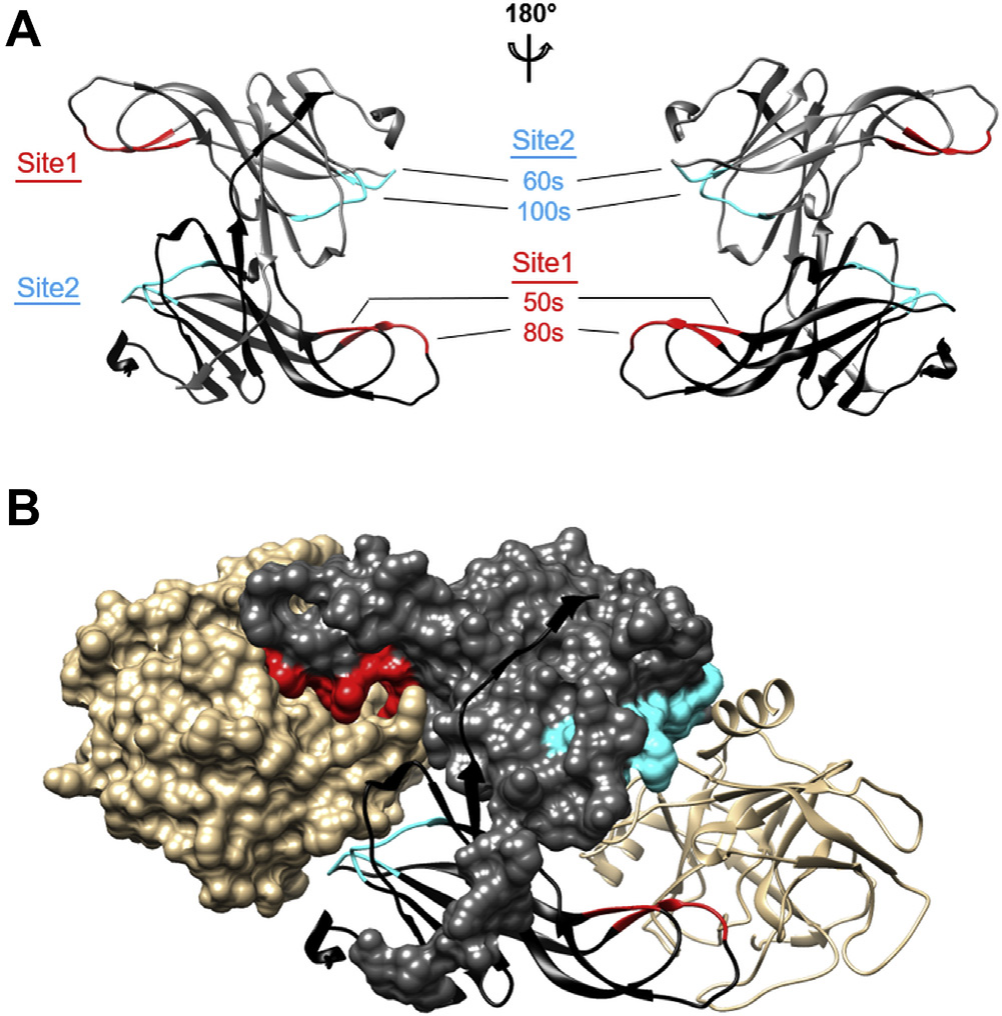
**The homodimeric structure and binding sites of ecotin.** Ecotin (in *gray*) has a twofold rotational symmetry and forms a stable homodimer, which can bind two proteases (in *beige*) simultaneously. The ecotin protomers contain two distinct protease-binding sites, each composed of two loops. The primary, that is, site1 binding site (*red*) is composed of the 80s loop, which represents the canonical inhibitory loop present in all reversible serine protease inhibitors, and the 50s loop, which supports the canonical loop conformation. The secondary, that is, site2 (*cyan*) binding site is composed of the 60s and 100s loops. *A*, in the homodimer ecotin, site1 of one protomer and site2 of the other one are positioned such that together can “chelate” the target proteinases. Because of the twofold symmetry, ecotin has two identical site1/site2 “chelators” and can simultaneously bind two proteinases. *B*, an ecotin:trypsin complex based on Protein Data Bank ID 1EZU (5) serves as an example for this unique arrangement.

**Figure 2:**
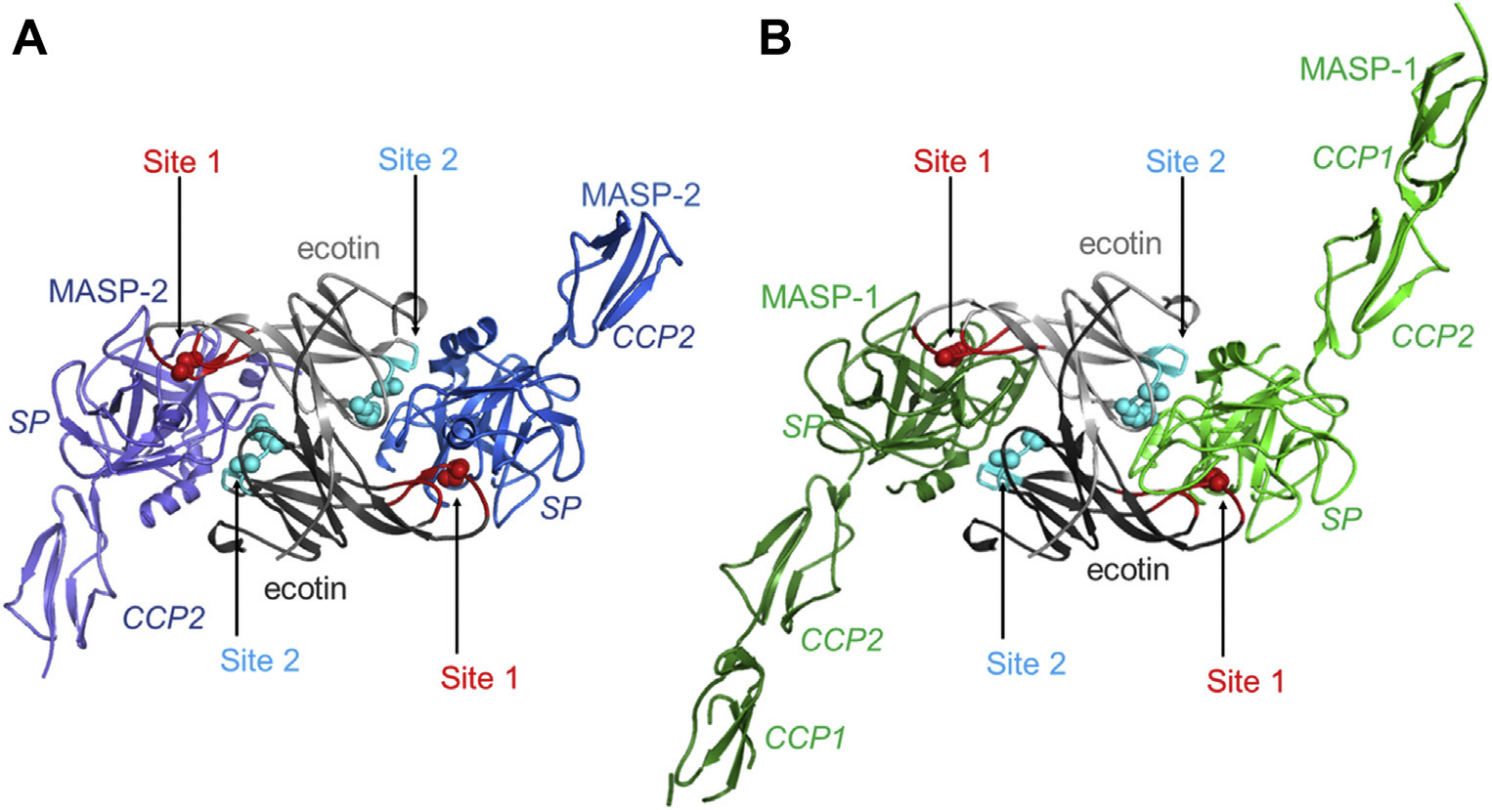
**The overall structure of the resolved complexes.** Both the MASP-2 (*CCP2–SP* fragment):ecotin (PDB ID: 7PQN) (*A*) and the MASP-1 (*CCP1–CCP2–SP* fragment):site1R ecotin (PDB ID: 7PQO) complexes (*B*) show a heterotetramer characteristic to ecotin. Ecotin protomers contact the SP domains of the MASPs through both of their binding surfaces, site1 (*red*) and site2 (*cyan*). Met84 (Arg84) of site1 and Arg108/Asn110 of site2 are shown as *spheres*. CCP, complement control protein; MASP, mannan-binding lectin-associated serine protease; PDB, Protein Data Bank; SP, serine protease.

The specificity of all reversible SP inhibitors, except ecotin, is governed largely by the nature of their canonical loop P1 residue (nomenclature of Schechter and Berger (6)), which enters the S1 specificity pocket of the enzyme. Inhibitors of trypsin-like enzymes have P1 Arg or Lys, whereas those of chymotrypsin-like enzymes Phe or Leu (7). In contrast, ecotin has a P1 Met, a highly flexible neutral residue capable of adapting to a variety of characteristically different S1 pockets. This P1 Met greatly contributes to the exceptional promiscuity of ecotin that inhibits the three main pancreatic enzyme types, trypsin, chymotrypsin, and elastase (1) and several highly specific plasma proteinases, such as fXa, fXIIa, plasma kallikrein, and leukocyte elastase (8, 9, 10). Yet, the suboptimal P1–S1 interaction of ecotin does not lead to a general low affinity, and this is due to the existence of site2 and the chelating mechanism of ecotin (4). The result is an extremely broad palette of target proteases, combined with tight binding of many different enzymes.

The relative importance of the P1 residue, the site2, and dimerization of ecotin were already quantitatively assessed in the context of the pancreatic enzymes and the urokinase-type plasminogen activator (11, 12, 13, 14, 15).

We have recently reported that *E. coli* ecotin inhibits all three MASPs of the complement system (16). While ecotin is a moderate MASP-1 inhibitor, it potently inhibits MASP-2 and MASP-3.

MASP-1 and MASP-2 are essential activators of the complement lectin pathway (LP) (17), whereas MASP-3 is the exclusive activator of pro-factor D (FD) in resting human blood, and therefore, it is essential for alternative complement pathway (AP) activation (18).

The three MASPs have unique structures and specificities, all have key physiologic functions and all are inhibited by ecotin. This prompted us to follow a previously outlined mutagenesis approach and decipher how site1, site2, and their structural preorganization *via* dimerization contributes to the inhibition of the three MASPs and blocking LP activation.

For testing the contribution of the P1 site, we generated an M84A (termed site1A) and an M84R (site1R) mutant. All three MASPs have trypsin-like specificity strictly preferring basic P1 residues (19). Therefore with site1R, we aimed to increase, whereas with the P1 truncating site1A, we aimed to decrease the affinity of ecotin toward these enzymes. For site2 weakening, we created the Arg108Ala/Asn110Ala (site2A) double mutant. These site2 positions were chosen based on the following considerations: (i) these residues are conserved among ecotin orthologs (10, 20), (ii) in a multiple-step directed evolution study, Arg108 remained absolutely, whereas Asn110 highly conserved (20), and (iii) both residues contact a variety of target proteases (21, 22). The aforementioned site1 and site2 mutations were also tested in combinations. The functional role of site1/site2 preorganization was tested by disrupting the homodimer structure of ecotin. This was achieved by removing the homodimer-stabilizing 10-aminoacid C-terminal arms yielding a stable monomeric ecotin form (13).

In parallel with the enzyme inhibition, LP inhibition, and size-exclusion chromatography–based oligomerization studies, we also solved the crystal structure of an MASP-2:ecotin and an MASP-1:site1R ecotin complex, which enabled structural interpretation of the functional results.

The functional analyses revealed that site1/site2 preorganization is essential for MASP-2 and MASP-3 inhibition and for blocking LP activation. With the point mutants, we show that individual contributions of site1 and site2 to MASP inhibition are characteristically enzyme dependent. The observed enzyme-specific differences can be congruently interpreted by our new MASP-1 and MASP-2:ecotin complex structures and by a previously reported MASP-3:ecotin structure of Gaboriaud *et al.* (22).

In this article, we show why MASP-2 and MASP-3 are inhibited strongly, whereas MASP-1 only weakly by ecotin. We also explain how ecotin uses its preorganized binding sites to achieve strong MASP-2 and MASP-3 inhibition in spite of the suboptimal P1 Met residue.

## Results and discussion

### Overall description of the MASP-2:ecotin and the MASP-1:site1R ecotin complex structures

The crystallization process is described in the Experimental procedures section. The crystal structure containing the activated CCP2–SP (complement control protein [CCP] domain; SP domain) fragment of MASP-2 in complex with ecotin was refined to a resolution of 2.4 Å. The structure is well defined by electron density (Table S1 and Fig. S1). The structure of the activated CCP1–CCP2–SP fragment of MASP-1 in complex with ecotin P1 M84R, that is, the site1R mutant, was solved and refined to a resolution of 3.4 Å. The core regions of the SP domain of MASP-1 and site1R ecotin as well as their binding regions stabilized by intermolecular interactions are well resolved in electron density (Table S1 and Fig. S2); in contrast, some loops and side chains on the molecular surface outside these regions are disordered and missing from the final structure (see Table S2 or a complete list of disordered residues). Both structures contain MASP:ecotin_2_:MASP heterotetramers (Fig. 2), in agreement with the results of Gaboriaud *et al*. (22), who reported a heterotetramer structure for the MASP-3:ecotin complex. All these MASP:ecotin complexes are typical in terms of simultaneously engaging both site1 (loops 50s and 80s) and site2 (loops 60s and 100s) of ecotin, without significant structural distortion of these ecotin loops. Importantly, ecotin does not contact the CCP domains.

**Figure 3:**
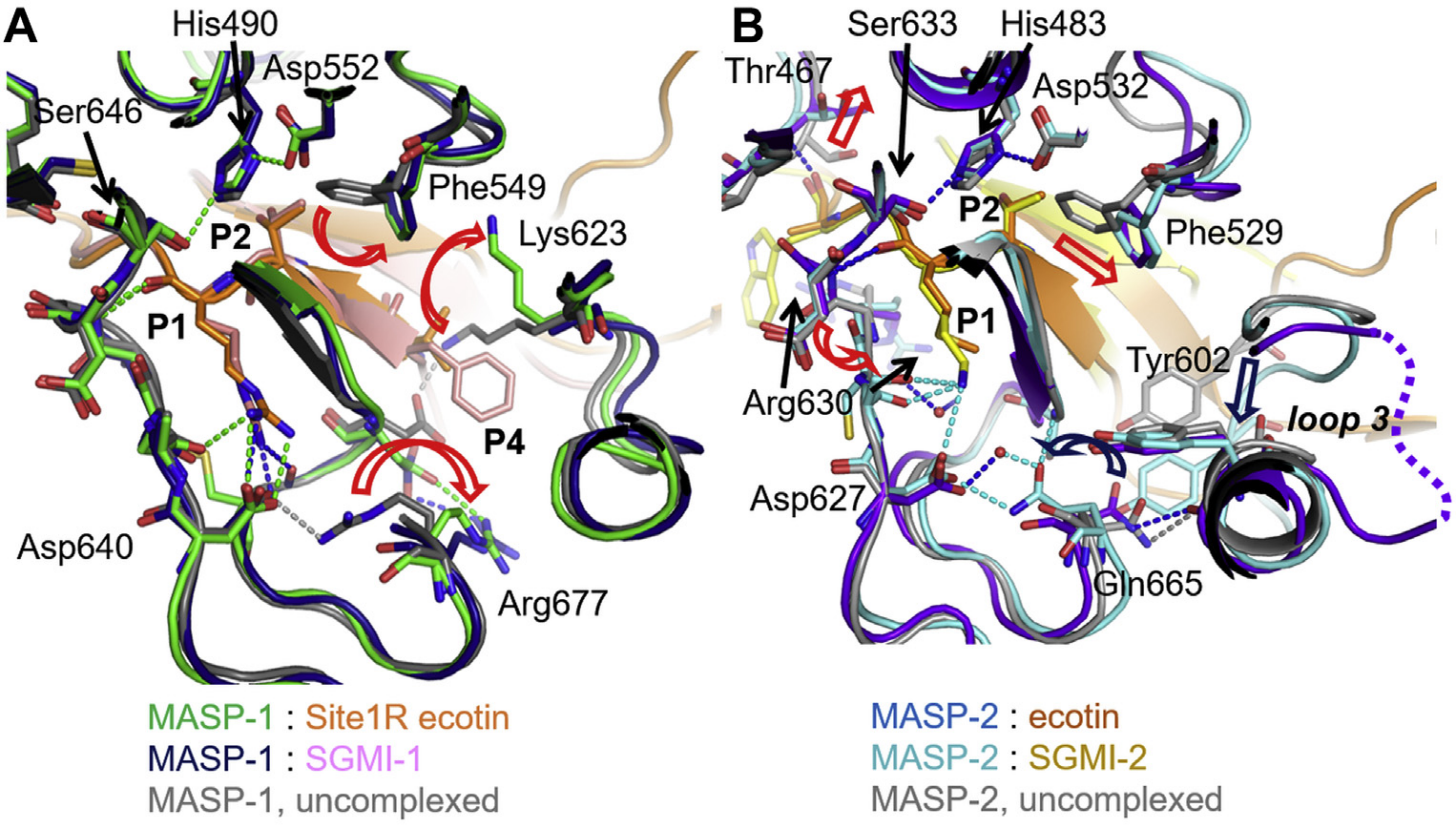
**Structural adaptation during the binding of inhibitors in MASP:ecotin and MASP:SGMI complexes.** Along with minor displacements, the Asp640 (Asp189c) of MASP-1 is released from the inner salt bridge to form a novel intermolecular salt bridge with the P1 Arg of site1R ecotin or of SGMI-1. This interaction is crucial for effective inhibition of MASP-1 (*A*). For MASP-2 binding, larger extent structural rearrangements are needed since the substrate-binding cleft of MASP-2 is restricted. Most arrangements are similar in the MASP-2:SGMI-2 and MASP-2:ecotin complexes, with one major difference: the *bottom* of the S1 pocket resembles an uncomplexed MASP-2 when complexed with ecotin as the S1–P1 interaction is suboptimal (*B*). Structures overlaid are as follows: *A*, uncomplexed MASP-1, PDB ID: 3GOV; MASP-1:SGMI-1 complex, PDB ID: 4DJZ; and MASP-1:site1R ecotin, PDB ID: 7PQO. *B*, uncomplexed MASP-2, PDB ID: 1Q3X; MASP-2:SGMI-2 complex, PDB ID: 3TVJ; and MASP-2:ecotin, PDB ID: 7PQN; respectively. MASP, mannan-binding lectin-associated serine protease; PDB, Protein Data Bank; SGMI, SGPI-derived MASP inhibitor.

In the MASP-2:ecotin and the MASP-1:site1R ecotin complexes, elements of the catalytic apparatus of the MASPs are in active arrangement, and site1 forms typical canonical interactions. Most structural changes are similar to those observed upon the *in vitro* evolved monospecific *Schistocerca gregaria* serine protease inhibitor-2 (SGPI-2)–based MASP inhibitors, SGPI-derived MASP inhibitor (SGMI) 1 binding to MASP-1 and SGPI-based MASP inhibitor 2 (SGMI-2) binding to MASP-2 (17, 23) (Fig. 3). While the P1 Arg in the MASP-1:site1R ecotin complex is in a fully extended conformation, the P1 Met in the MASP-2:ecotin complex does not fully occupy the S1 pocket. Interestingly, the bottom of the S1 pocket of MASP-2 resembles that of an uncomplexed MASP-2 (24) rather than an MASP-2:SGMI-2 complex, in which the S1 pocket is occupied by a P1 Lys. Apparently, rotation of the Gln665 side chain to form a hydrogen bond network around the bottom of the S1 pocket is characteristic to MASP-2 complexes where the S1–P1 salt bridge is established (MASP-2:SGMI-2 complex; Fig. 3*B*).

A comparison of site1 interactions of MASP-1:site1R ecotin, MASP-2:ecotin, and MASP-3:ecotin complexes (the latter presented by Gaboriaud *et al.* (22) [PDB ID: 4IW4]) reveal that while the same set of backbone hydrogen bonds is present for the P4–P1 core region in all three complexes, the contact region of the 80s loop of ecotin extends to the S7–S4′ region in the substrate-binding grooves of MASP-1 and MASP-3, whereas it is somewhat shorter for MASP-2 (Fig. S3, *A*–*C*). The 80s loop backbone is accommodated in very similar conformations in the three complexes (Fig. S3*D*).

### Site1 and site2 point mutations do not alter the homodimer structure of ecotin

Five ecotin point mutants were produced to decipher the contribution of each binding site of ecotin to the inhibition of the MASPs. Site1R ecotin contains a P1 M84R, whereas site1A an M84A mutation. Site2A carries the R108A/N110A double mutation. We also made two combined variants, site1R/site2A and site1A/site2A.

In a size-exclusion chromatography analysis, all five mutants had the same elution volume as WT ecotin verifying that these replacements do not alter the original homodimer structure of the inhibitor (Fig. S4*A*).

Monomeric ecotin variants (13) were clearly separated from the five dimeric point mutants by size-exclusion chromatography (Fig. S4, *A* and *B*).

### Functional importance of site1 is highest on MASP-1, medium on MASP-2, and lowest on MASP-3

Equilibrium inhibitory constant (*K*_I_) and calculated standard free energy (ΔG^0^), that is, “binding energy” values of ecotin variants against the three MASP enzymes are listed in Table 1.

**Table 1:** Equilibrium inhibitory constant and calculated binding free energy values of point mutant ecotin variants on the three MASPs

Ecotin variant	MASP-1		MASP-2		MASP-3	
	*K*_I_ (M)	ΔG^0^ (kJ/mol)	*K*_I_ (M)	ΔG^0^ (kJ/mol)	*K*_I_ (M)	ΔG^0^ (kJ/mol)
WT^a^	4.2 ± 0.3 × 10^−6^	30.7 ± 0.2	1.1 ± 0.1 × 10^−8^	45.4 ± 0.3	5.0 ± 0.2 × 10^−10^	53.1 ± 0.1
Site1R	6.0 ± 1.4 × 10^−10^	52.6 ± 0.6	4.0 ± 1.0 × 10^−11^	59.3 ± 0.7	3.0 ± 1.0 × 10^−11^	60.0 ± 1.0
Site1A	ND	ND	1.4 ± 0.1 × 10^−6^	33.4 ± 0.1	5.1 ± 0.5 × 10^−9^	47.3 ± 0.2
Site2A	ND	ND	9.8 ± 0.2 × 10^−7^	34.3 ± 0.0^b^	8.8 ± 1.3 × 10^−8^	40.2 ± 0.4
Site1R/site2A	2.4 ± 0.1 × 10^−9^	49.1 ± 0.1	1.8 ± 0.3 × 10^−9^	49.8 ± 0.4	6.1 ± 0.7 × 10^−9^	46.9 ± 0.3
Site1A/site2A	ND	ND	ND	ND	ND	ND

Abbreviation: ND (no ΔG° value could be calculated because the binding energy of the mutant was below the estimated detection limit of 24.5 kJ/mol).

Ecotin P1 replacement alters binding affinity in an MASP-dependent manner. The effect is largest on MASP-1, medium on MASP-2, and smallest on MASP-3 (ΔΔG values are listed in Table 2). In combination with the affinity-improving site1R mutation, simultaneous perturbation of the conserved site2 residues R108 and N110 by Ala replacement shows the opposite trend, affecting the interaction with MASP-3 at the greatest, MASP-2 at medium, and MASP-1 at the lowest level (ΔΔG values are listed in Table 3). Data are the average of at least two independent measurements ± SD.

a Data from previous study (16).

b SD is lower than 0.1 kJ/mol.

Ecotin binds MASP-1, MASP-2, and MASP-3 with 30.7, 45.4, and 53.1 kJ/mol binding energy, respectively. Our estimated detection limit for inhibitory constant–based binding energy is 24.5 kJ/mol (see the Experimental procedures section for explanation). Note that the binding energy of the ecotin–MASP-1 interaction is close to this limit.

**Figure 4:**
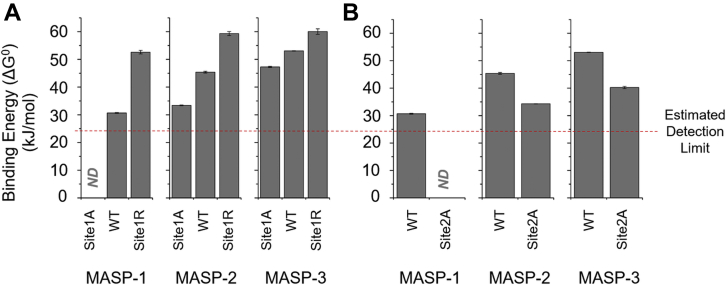
**Binding free energy (ΔG°) values of site1 and site2 ecotin variants toward the three MASP enzymes.***A*, site1 mutations cause the greatest binding free energy (*i.e.*, binding energy) changes for MASP-1, the smallest ones for MASP-3, whereas MASP-2 is in between. In the case of site2 (*B*), the greatest binding energy change is observed for MASP-3, closely followed by MASP-2. As MASP-1 binds inherently weakly to ecotin, the binding energy change for site2 weakening could not be determined. Results are the average of at least two measurements, the SD shown by the error bars. ND, no ΔG° value could be calculated because the binding energy of the mutant was below the estimated detection limit of 24.5 kJ/mol. MASP, mannan-binding lectin-associated serine protease.

The M84A replacement removes the original P1 side-chain atoms beyond the beta carbon. In terms of inhibitory constant, this alteration causes a 126-fold decrease in the affinity of ecotin toward MASP-2 and a 10-fold decrease toward MASP-3, corresponding to binding energy changes (ΔΔG) of −12 and −5.8 kJ/mol, respectively (Tables 1 and 2; Fig. 4). However, for the weak MASP-1:ecotin interaction, the resulting binding energy value for the site1A variant falls below the 24.5 kJ/mol estimated detection limit.

**Table 2:** Calculated binding free energy changes caused by the P1 replacement of ecotin

Ecotin variants compared	MASP-1	MASP-2	MASP-3
	ΔΔG (kJ/mol)		
Site1R *versus* WT	+21.9	+13.9	+6.9
Site1A *versus* WT	ND	−12.0	−5.8

Abbreviation: ND (no ΔΔG value could be calculated because the binding energy of the mutant was below the estimated detection limit of 24.5 kJ/mol).

Standard free energy (ΔG^0^) (*i.e.*, binding energy) values of site1 mutants from Table 1 were compared with that of the WT to calculate ΔΔG values. A positive value indicates an increase of affinity, whereas a negative one indicates a decrease. ΔΔG is calculated from the average ΔG^0^ values in Table 1.

All three MASPs are trypsin-like enzymes having a negatively charged S1 pocket that prefers positively charged Lys or Arg P1 (19). In agreement with this, M84R replacement increased binding affinity to MASP-1, MASP-2, and MASP-3 7000-fold, 275-fold, and 17-fold, respectively (Fig. 4, Tables 1 and 2), corresponding to calculated ΔΔG values 21.9, 13.9, and 6.9 kJ/mol, respectively.

For MASP-2 and MASP-3, affinities against both the M84A and the M84R variants could be determined. Notably, for these enzymes, the M84A replacement yields about the same extent of affinity drop as the affinity is increased by the M84R change: −12.0 kJ/mol *versus* 13.9 kJ/mol for MASP-2 and −5.8 kJ/mol *versus* 6.9 kJ/mol for MASP-3 (Table 2). If the same trend applies for MASP-1, the binding energy decrease upon the M84A mutation would be approximately −20 kJ/mol.

These data reveal that the role of P1 and therefore site1 is markedly enzyme dependent, being crucial for MASP-1, substantial for MASP-2, and significantly lower for MASP-3. The observed phenomenon can be explained through the crystallographic data.

MASP-1 has a readily accessible substrate-binding groove (25). Upon ecotin binding, only minor displacements of residues are observable on the side of the enzyme, including Phe549 (c99, with chymotrypsinogen numbering), loop 3 (labeled as defined by Perona and Craik (26)), and Lys623 (c174). However, in the free enzyme, an intramolecular salt bridge is formed between the Asp640 (Asp c189, a key residue defining the specificity of the S1 pocket) and the Arg677 (c224) (23, 25) (Fig. 3*A*). Our crystal structure demonstrates that site1R ecotin completely disrupts this self-inhibited ground state by a salt bridge exchange mechanism. Such a salt bridge seems to be crucial for effective inhibition of MASP-1, regardless of the inhibitor family; as in previous directed evolution studies, MASP-1 exclusively selected a P1 Arg residue when the sunflower trypsin inhibitor–based sunflower MASP inhibitor 1 (SFMI-1) or the SGPI-based MASP inhibitor 1 was developed (Fig. 3*A*) (17, 27). Besides the P1–S1 salt bridge, the P1 Arg forms additional H-bonds as well. The P1 Met of WT ecotin or the Ala of the site1A form cannot achieve the same result. However, unlike a P1 Ala, the long and flexible WT P1 Met residue could at least accommodate to the distorted S1 pocket and contribute to the overall binding energy. There is another structural feature that could also contribute to the enhanced role of the P1 residues in MASP-1 inhibition. Outside the S1 pocket, but within the site1 region, ecotin has a less favorable interaction with MASP-1 than with MASP-2. Namely, upon complex formation, ecotin Arg54 binds in the vicinity of a bulky positively charged segment of MASP-1. In all, these structural phenomena explain the low MASP-1 inhibitory potency of WT ecotin and the relatively high importance of the P1 residue.

For MASP-2, an enzyme with a more restricted active-site access, the binding of an inhibitor requires larger conformational changes, similarly to those of described for the MASP-2:SGMI-2 complex, with SGMI-2 bearing a Lys at P1 (PDB ID: 3TVJ (23)) (Fig. 3*B*). These changes involve Arg630, Thr466–Thr467, Phe529, and loop 3, with the 603 to 610 segment being destabilized. A flexible P1 Met can fit into the S1 pocket at a lower energetic cost; however, it cannot fully occupy the pocket, the contact remains suboptimal, as in the case of the MASP-3:ecotin complex (22) (Fig. S3). A P1 Arg can establish stronger interactions with the S1 pocket of MASP-2; however, accommodating a P1 Arg requires larger conformational changes from the enzyme, than accommodating a Met. In all, although in terms of electrostatic complementarity, a P1 Arg is a better fit for the negatively charged S1 pocket, there are some energetic costs that lower the energetic gain of the M84R replacement.

In the case of MASP-3, the situation is similar. The S1 pocket-lining loop 1 and loop 2 are slightly distorted, and the accommodation of P1 is not perfect, weakening the interaction between MASP-3 and the 80s loop of ecotin (Fig. S3). This could explain why P1 replacements cannot exert a dramatic effect as in classical canonical SP inhibition models, where a lock and key binding mechanism dominates (28, 29).

### Functional importance of site2 is lowest on MASP-1, medium on MASP-2, and highest on MASP-3

Simultaneous Ala replacement at site2 positions Arg108 and Asn110 was tested in the context of both P1 Met (WT) and P1 Arg (site1R) ecotin. In both contexts, the site2A mutation weakens interactions toward all three MASPs but to an enzyme-dependent extent (Tables 1 and 3).

**Table 3:** Calculated binding free energy contributions of secondary binding site amino acid replacements in the context of WT and site1R ecotin

Ecotin variants compared	MASP-1	MASP-2	MASP-3
	ΔΔG (kJ/mol)		
Site2A *versus* WT	ND	−11.1	−12.9
Site1R/site2A *versus* site1R	−3.5	−9.4	−13.1

Abbreviation: ND (no ΔΔG value could be calculated because the binding energy of the mutant was below the estimated detection limit of 24.5 kJ/mol).

Standard free energy (ΔG^0^) (*i.e.*, binding energy) values of ecotin mutants from Table 1 were compared to calculate ΔΔG values. The negative values represent a decrease of affinity. ΔΔG is calculated from the average ΔG^0^ values in Table 1.

WT ecotin is already a weak inhibitor of MASP-1, and site2A mutations drop its affinity below our estimated 24.5 kJ/mol detection limit. This suggests that the affinity drop of site2A on MASP-1 exceeds 6.2 kJ/mol. Affinity drops against the high-affinity binders MASP-2 and MASP-3 were measurable and found to be 11.1 kJ/mol (88-fold *K*_I_ change) and 12.9 kJ/mol (176-fold *K*_I_ change), respectively (Table 3).

In the P1 Arg context, the site2A mutation caused 3.5 kJ/mol affinity decrease (fourfold *K*_I_ change) on MASP-1, 9.4 kJ/mol (45-fold *K*_I_ change) on MASP-2, and 13.1 kJ/mol (200-fold *K*_I_ change) on MASP-3.

All in all, compared with the case of site1, a reciprocal trend is observed regarding the contribution of site2 of ecotin to the inhibition of the three MASPs.

The crystallographic data obtained from the MASP-1:site1R ecotin and the MASP-2:ecotin complexes, and the data from the studies of Gaboriaud *et al.* (22), largely explain the functional consequences of the site2A mutation. Site2 of ecotin consists of loop 60s (segment 63–70 forming a mostly conserved hydrogen bond pattern) and loop 100s (108–112 forming a conserved hydrogen bond through Asn110 and variable interactions through Arg108 and Lys112). Arg108 is a contact position for several target proteases having different specificities (3, 21, 22).

**Figure 5:**
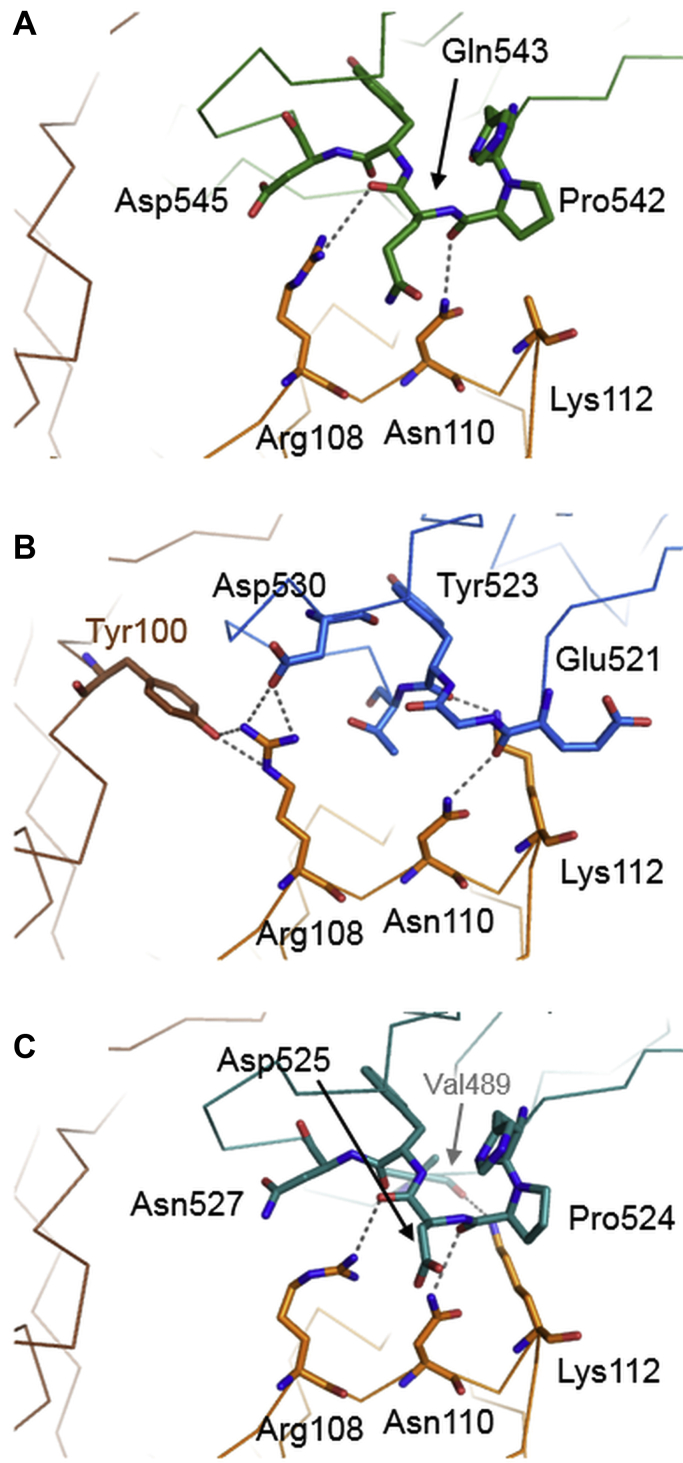
**Interactions of ecotin site2 with the three MASP enzymes.** Loop 100s of ecotin site2 forms multiple interactions with the three enzymes (MASP-1: *green*, MASP-2: *blue*, and MASP-3: *turquoise*). Importantly, while Asn110 forms identical interactions with all MASPs, the Arg108 interactions are enzyme specific, showing a transient H-bond with MASP-1 (*A*). In contrast, it forms stable interactions with MASP-2 (*B*) (extended H-bonding network) and MASP-3 (*C*) (shorter H-bonding distance). While ecotin Lys112 does not contact MASP-1, it forms H-bonds with MASP-2 and MASP-3. The structures suggest that replacing Arg108 and Asn110 with Ala in site2A variant disrupts only weak interactions with MASP-1 but strong interactions with MASP-2 and MASP-3 (possibly also destabilizing Lys112 interactions). This is in perfect line with the measured larger binding energy loss caused by site2A for MASP-2 and MASP-3 compared with MASP-1, in which neither Arg108 nor Lys112 establishes stable interactions with the enzyme (*A*). See also Fig. S5 and Table S3 for a broader overview and a complete list of the contacts. MASP, mannan-binding lectin-associated serine protease.

At site2, MASP-2 and MASP-3 ecotin complexes reveal more extensive binding surfaces with more hydrogen bonds than MASP-1 (Figs. 5, S5 and Table S3). Comparison of the interactions of the Arg108–Asn110–Lys112 motifs reveals characteristic differences in MASP complexes. In the MASP-1 complex, the H-bond formed by Lys112 is missing, and a larger number of disordered side chains are observed indicating greater flexibility and weaker interactions. In MASP-3, Asp525 and Asn527 orientate the Arg108 of ecotin to establish a bond with the carbonyl group of Asp525 (Fig. 5*C*). Such an effect is missing from MASP-1, where Asp545 and Gln543 do not assist orientation of Arg108; only a transient bond is present between Arg108 and Gln543, and a weak electrostatic interaction exists with Asp545 (Fig. 5*A*). In the MASP-2 complex, ecotin Arg108 forms a unique salt bridge with Asp530 of the enzyme and an additional H-bond with Tyr100 of the other ecotin protomer (Figs. 5*B* and S5). Ecotin Asn110 contacts Pro542 of MASP-1, Glu521 of MASP-2, and Pro524 of MASP-3. It also stabilizes the 100s loop, therefore enabling Lys112 and Leu113 to establish contacts with the target enzyme: Lys112 contacts Tyr523 and Val489 of MASP-2 and MASP-3, respectively, whereas such contacts are missing from the MASP-1:site1R ecotin complex (Fig. 5).

In all, Arg108 and Asn110 have extensive interactions with MASP-2 and MASP-3. While MASP-1 also engages Arg108 and Asn110, it has fewer and apparently less stable interactions with ecotin site2; the loss of contacts through the site2A mutation has greater negative effect on the affinity toward MASP-2 and MASP-3 than toward MASP-1.

### Simultaneous weakening of site1 and site2 abolishes MASP-inhibitory potency of ecotin

As we have shown, the MASP-1:ecotin complex is so unstable that perturbing it by Ala mutations at either site1 or site2 completely abolishes MASP-1 inhibition. In contrast, MASP-2 and MASP-3, which bind ecotin with substantially higher affinity, are inhibited both by site1A and site2A ecotin variants. Nevertheless, when both sites are weakened in the site1A/site2A variant, ecotin becomes nonfunctional against all three MASP enzymes (Table 1).

### Effects of site1R and site2A alterations are largely additive

As listed in Table 3, for MASP-2 and MASP-3, the effects of the site2A mutations are rather context independent, that is, are similar with P1 Met and P1 Arg site1 background. The context-dependent differences are only 1.7 kJ/mol for MASP-2 and 0.2 kJ/mol for MASP-3. For MASP-1, in P1 Arg context, the site2A effect is 3.5 kJ/mol, whereas in P1 Met context, it could only be estimated to be at least 6.2 kJ/mol. This does not rule out additivity but leaves the case open for some cooperativity.

### Site1 and site2 apparently act synergistically against MASP-3

If site1A and site2A mutations would exert their effects independently, the observed affinity drop in the combined site1A/site2A variant should be a simple sum of the individual site1A-driven and site2A-driven affinity drops. This sum for MASP-1 cannot be calculated as site1A ecotin is practically inactive on MASP-1.

For MASP-2, this sum yields a 23.1 (12.0 + 11.1) kJ/mol affinity drop that then would result in a 22.3 (45.4–23.1) kJ/mol binding energy. Note that the weakest inhibition we could detect corresponds to a calculated 24.5 kJ/mol binding free energy. It means that in the case of additivity, MASP-2 inhibition by site1A/site2A ecotin should be undetectable, which is what we found. This means that additivity might apply for MASP-2, although positive cooperativity of site1A and site2A cannot be ruled out.

For MASP-3, the sum of affinity drops is 18.7 (5.8 + 12.9) kJ/mol that would result in 34.4 (53.1–18.7) kJ/mol binding energy. This value is 9.9 kJ/mol higher than our detection limit and would be measurable. Yet, site1A/site2A ecotin does not inhibit MASP-3 to a measurable extent (Table 1). This suggests substantial positive cooperativity, that is, site1 and site2 in WT ecotin strengthen each other. Weakening any of them by Ala mutation weakens the function of the other nonmutated site as well.

Eggers *et al.* (14) detected a similar phenomenon regarding the energetic contribution of the ecotin-binding sites interacting with rat trypsin.

### Native preorganization of the binding site network of ecotin is a key for MASP-2 and MASP-3 inhibition

We found that for efficient overall MASP-inhibitory activity, both site1 and site2 have important, albeit MASP-specific roles. Next, we tested the functional importance of the spatial preorganization of site1 and site2 provided by the homodimer ecotin structure. C-terminally shortened ecotin variants are monomers and can still form the same type of heterotetramer enzyme–inhibitor complex as WT ecotin (13). It is because they still retain their intact site1 and site2, and through these can still associate with their target enzymes. An important difference is that site1 and site2 are no longer structurally preorganized for simultaneous protease binding in monomeric ecotin. If both site1 and site2 of ecotin contribute to the binding free energy, and their WT prearrangement allows for simultaneous binding, monomerization should lead to a decrease of binding affinity. We aimed to determine the extent of this expected binding free energy drop and see whether it is also enzyme dependent.

Using monomeric WT and site1R ecotin variants, we tested how removal of the C-terminal arm affects binding affinity on the three MASPs. The corresponding equilibrium inhibitory constants and calculated binding energies are listed in Table 4, whereas the calculated energetic contribution of binding site preorganization is shown in Table 5.

**Table 4:** Equilibrium inhibitory constant and calculated binding free energy values of dimer and monomer ecotin variants on the three MASP enzymes

Ecotin variant	MASP-1		MASP-2		MASP-3	
	*K*_I_ (M)	ΔG^0^ (kJ/mol)	*K*_I_ (M)	ΔG^0^ (kJ/mol)	*K*_I_ (M)	ΔG^0^ (kJ/mol)
WT^a^	4.2 ± 0.3 × 10^−6^	30.7 ± 0.2	1.1 ± 0.1 × 10^−8^	45.4 ± 0.3	5.0 ± 0.2 × 10^−10^	53.1 ± 0.1
Monomer	2.4 ± 0.1 × 10^−5^	26.4 ± 0.1	ND	ND	ND	ND
Site1R	6.0 ± 1.4 × 10^−10^	52.6 ± 0.6	4.0 ± 1.0 × 10^−11^	59.3 ± 0.7	3.0 ± 1.0 × 10^−11^	60.0 ± 1.0
Site1R monomer	1.4 ± 0.1 × 10^−8^	44.8 ± 0.3	4.1 ± 0.2 × 10^−8^	42.1 ± 0.1	1.7 ± 0.0 × 10^−7^^b^	38.6 ± 0.0^c^

Abbreviation: ND, no inhibition is detected as the binding free energy of the mutant was below the estimated detection limit of 24.5 kJ/mol.

In the context of WT P1 Met, preorganization of the two binding sites is essential for inhibiting MASP-2 and MASP-3. Data are the average of at least two independent measurements ± SD.

a Data from previous study (16).

b SD is lower than 0.1 × 10^−7^ M.

c SD is lower than 0.1 kJ/mol.

**Table 5:** Energetic contribution of primary and secondary binding site preorganization

Ecotin variants compared	MASP-1	MASP-2	MASP-3
	ΔΔG (kJ/mol)		
Monomer *versus* WT	−4.3	ND	ND
Site1R monomer *versus* site1R	−7.8	−17.2	−21.4

Abbreviation: ND (no ΔΔG value could be calculated because the binding free energy of the mutant was below the estimated detection limit of 24.5 kJ/mol).

Difference of calculated ΔG^0^ values (from Table 4) is shown for cases where the inhibition constants could be determined.

The data clearly show that for P1 Met ecotin, the preorganized binding sites are essential for MASP-2 and MASP-3 inhibition, as monomer ecotin is unable to inhibit these enzymes to measurable level. In contrast, while MASP-1 is able to utilize the preorganized sites, this contribution is small, that is, inhibition through the monomeric Met84 form represents only 4.3 kJ/mol binding affinity loss (Table 5) and remains measurable.

The site1R mutation increases binding affinity of the monomeric ecotin for all MASPs and brings the affinity to a measurable level on MASP-2 and MASP-3. Therefore, in the context of site1R, the contribution of the preorganized binding sites could be calculated for all three MASPs as listed in Table 5. The data demonstrate that preorganization of site1 and site2 is significantly more important for MASP-2 and MASP-3 than for MASP-1, which is in line with the observed greater contribution of site2 to binding MASP-2 and MASP-3 as opposed to binding MASP-1.

Moreover, if binding energy contribution of site1/site2 preorganization would be independent of the P1 residue for MASP-2 and MASP-3, monomer P1 Met ecotin would bind MASP-2 with 28.2 (45.4–17.2) kJ/mol affinity and MASP-3 with 31.7 (53.1–21.4) kJ/mol affinity. As both values exceed the 24.5 kJ/mol detection limit, both would be measurable. This demonstrates that for the P1 Met ecotin, binding site preorganization is even more important for MASP-2 and MASP-3 than for the P1 Arg ecotin. Notably, the opposite is observed for MASP-1, where P1 Arg slightly increases the contribution of preorganization.

### Quaternary structures of MASP-inhibiting dimer and monomer ecotin variants

To build a congruent mechanistic model for MASP:ecotin interactions, for all ecotin–MASP pairs having high enough binding affinity to keep the complex stable during the time of the experiment, we assessed their quaternary structure by size-exclusion chromatography.

**Figure 6:**
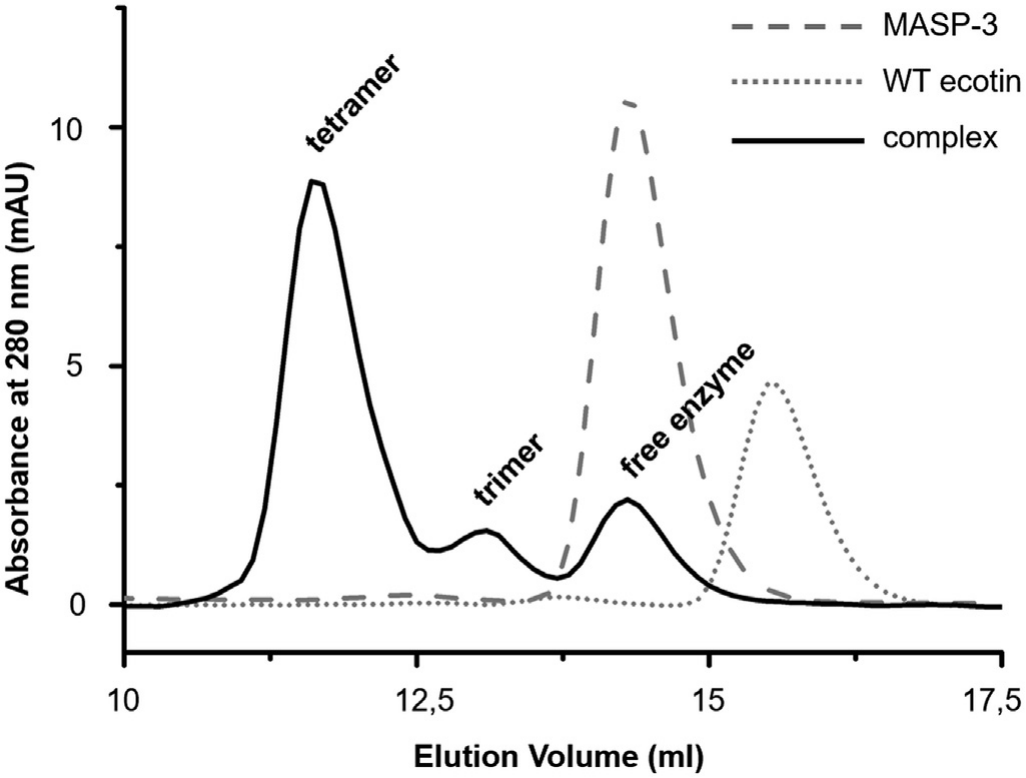
**Size-exclusion chromatography of inner control proteins and their complexes.** In three separate runs, WT ecotin (which is a stable homodimer), or MASP-3, or WT ecotin mixed with an excess of MASP-3 was loaded on the Superdex 200 column. In the chromatogram of the enzyme:ecotin complex, the third peak corresponds to free MASP-3, whereas the first peak corresponds to heterotetramer (MASP-3)_2_:ecotin_2_ complex. Between the two peaks, an MASP-3:ecotin_2_ heterotrimer is detected. MASP, mannan-binding lectin-associated serine protease.

Although in this article we present two heterotetramer crystal structures, one for WT ecotin formed with MASP-2, and another for site1R ecotin formed with MASP-1, we used the first published (MASP-3)_2_:ecotin_2_ complex as an inner heterotetramer control (22). The size-exclusion gel properly separated the following components: the heterotetramer and heterotrimer complexes, dimer ecotin, and the free enzymes (Fig. 6).

The peaks in Figure 6 demonstrate that when WT ecotin is oversaturated with MASP-3, dominantly heterotetramers are formed, but a small amount of heterotrimer is also detectable. The presence of heterotrimer, that is, dimer ecotin binding to only one enzyme in spite of the stoichiometric excess of the enzyme implies that the enzyme–inhibitor interaction is slightly stronger in the trimer than in the tetramer, that is, binding a second enzyme slightly weakens the interaction of both enzymes with ecotin, probably because ecotin structure might need to go through some conformational change.

**Figure 7:**
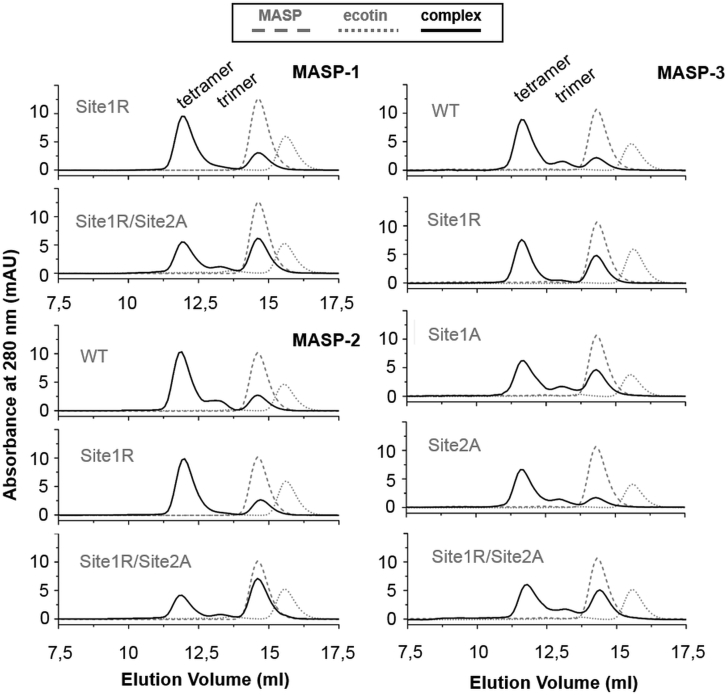
**Complexes of the three MASPs with dimeric ecotin variants.** For all MASP:inhibitor pairs, the dominant form is the heterotetramer, comprising two MASP enzymes and an ecotin homodimer. In most cases, a peak representing the heterotrimer is also present. Note that the highest affinity complexes (data listed in Table 1) involving site1R ecotin variant yield the lowest proportion of heterotrimers. MASP, mannan-binding lectin-associated serine protease.

Regarding the heterotrimer proportion in Figure 7, there is a clear trend for all MASPs. While samples containing the highest affinity site1R ecotin contain only hardly detectable amounts of heterotrimer complexes, all other weaker complexes generate a distinct heterotrimer peak. Apparently, when site1R is combined with WT site2 in a dimer ecotin context, the P1 Arg residue provides enough extra binding free energy that fully covers the energetic cost of accommodating two enzymes simultaneously, resulting in an enhanced stability heterotetramer. Results of the size-exclusion chromatography are in accordance with our crystal structures, showing that the dominant form of the MASP:ecotin complexes is the heterotetramer.

**Figure 8:**
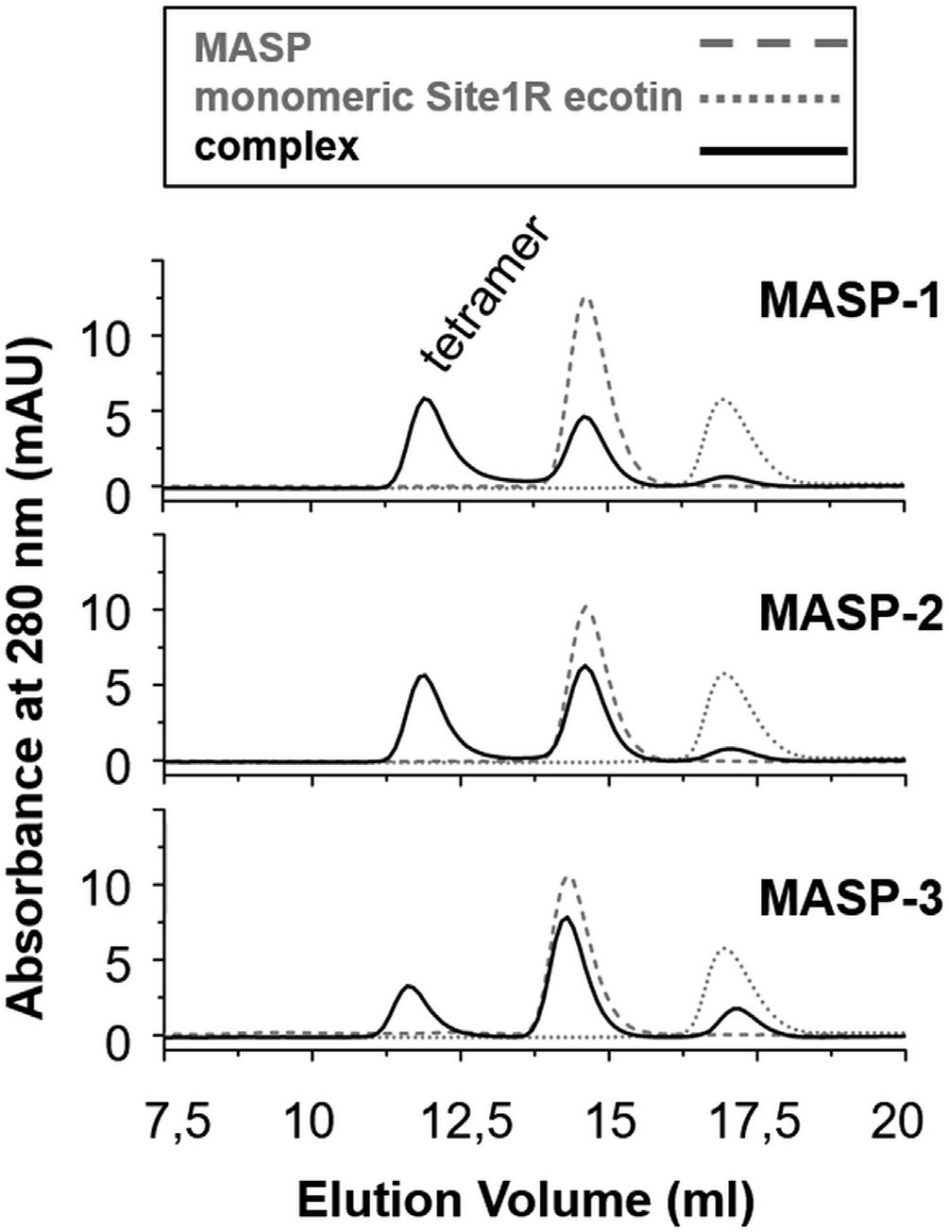
**Monomeric site1R ecotin forms only heterotetramer complexes with the three MASP enzymes.** A characteristic peak of heterotetramer MASP:ecotin complexes appears on each chromatogram, at the 11.25 to 12.5 ml elution volume interval. No complexes of lower oligomerization levels were detected, showing that only the heterotetramer form of the MASP:site1R monomer ecotin is stable. No heterotrimers were found, indicating that association exclusively through the site2 of ecotin is unlikely; such complexes lack the required stability to be detected by chromatographic methods. MASP, mannan-binding lectin-associated serine protease.

We tested two monomeric ecotin versions, one having WT P1 Met and the other P1 Arg. We found that only the site1R monomer provided stable enough complexes for size-exclusion chromatography studies. The complexes formed between monomeric site1R ecotin and MASPs were pure heterotetramers with no sign of any heterotrimers (Fig. 8). This suggests that when a site1R monomer ecotin and an MASP enzyme associate, the heterodimers quickly associate forming more stable heterotetramers. Such association engages two WT site2 regions simultaneously. Whenever a heterotetramer loses any component, it results in a metastable and therefore undetectable trimer.

### Toward a congruent model of the MASP-dependent binding mechanism of ecotin

The MASP-1:ecotin complex is unstable: weakening the ecotin either through a site1A or a site2A mutation renders the interaction undetectably weak. Yet, monomerization of ecotin results in only a small effect. The case for MASP-2 and MASP-3 is the exact opposite: site1A or site2A mutation can only weaken the interaction, whereas monomerization of ecotin completely abolishes it. The results obtained on MASP-specific individual ecotin-binding site contributions combined with data on the quaternary structure of the MASP:ecotin complexes led us to a congruent binding mechanism model as discussed later here.

For MASP-1, the interaction through ecotin site1 is suboptimal since it does not disrupt the self-inhibited state of the S1 pocket (Figs. 3 and S3). However, as the substrate-binding groove is readily accessible and the complex can form without significant conformational changes, this interaction can be a quick single-step process. In the WT ecotin dimer, the weak and apparently transient site2 interaction can instantly contribute with just enough energy to render the inhibition measurable. With monomer ecotin, the same site1 interaction through open binding grooves yields heterodimers, which associate through simultaneously engaging two secondary binding sites (site2) and form a stable heterotetramer. This second step can also be quick as the site2 interaction does not require significant conformational adaptation.

In contrast, for MASP-2 and MASP-3, ecotin monomerization abolishes complex formation, in spite of the observed large contribution of site2 to binding of both enzymes. The only comprehensive explanation is that these enzymes cannot form a stable site1 interaction with monomeric ecotin. This is in perfect line with the notion that site1 interaction requires significant conformational changes on the side of both MASP-2 and MASP-3 (Figs. 3 and S3), and therefore, the interaction is a multistep process, where the first transient complexes are of very low affinity. For such a slow-binding–induced fit process, site2 needs to exert a stabilizing effect from the beginning through a relatively strong interaction. Our proposed model suggests that upon association, WT homodimeric ecotin can engage site1 and site2 either simultaneously or in any order, depending on binding site availability on the protease. The two extremes of this model would be the “site1-first” and the “site2-first” scenario. We note that for the MASP-3:ecotin interaction, Gaboriaud *et al.* (22) already mentioned a model equivalent to our “site2-first” scenario.

In the case of MASP-3, there are further evidences implying that its substrate-binding site requires significant conformational changes for the binding of substrate-like inhibitors. We already reported that ecotin inhibits MASP-3 by a slow binding mechanism suggesting a slow structural maturation of the complex (16). We also reported that our *in vitro* evolved MASP-3 inhibitor, TFMI-3, which binds only through a canonical, that is, primary binding site, has an unusually slow association rate (18), suggesting a mechanism requiring conformational changes in the binding site of MASP-3. Furthermore, MASP-3 is the only MASP that has no natural serpin inhibitor. Serpins are irreversible protease inhibitors that need to be cleaved for covalent acyl enzyme complex formation, and this mechanism relies on a readily available stable substrate-binding cleft of the enzyme (30). Apparently, MASP-3 lacks such an accessible binding groove.

### The revealed cooperative effects further support the inhibitory mechanism model

Site1R ecotin containing the Met84Arg mutation is a general high-potency inhibitor of all three MASP enzymes. The elevated stability of the corresponding complexes allowed for estimating the extent of site2 and site1/site2 preorganization contribution for MASP-2 and MASP-3 binding as well, where in the WT site1 context, these were not assessable (Table 5). Unlike the original P1 Met form of monomer ecotin, which inhibits only MASP-1, its site1R version is active on all three enzymes.

**Figure 9:**
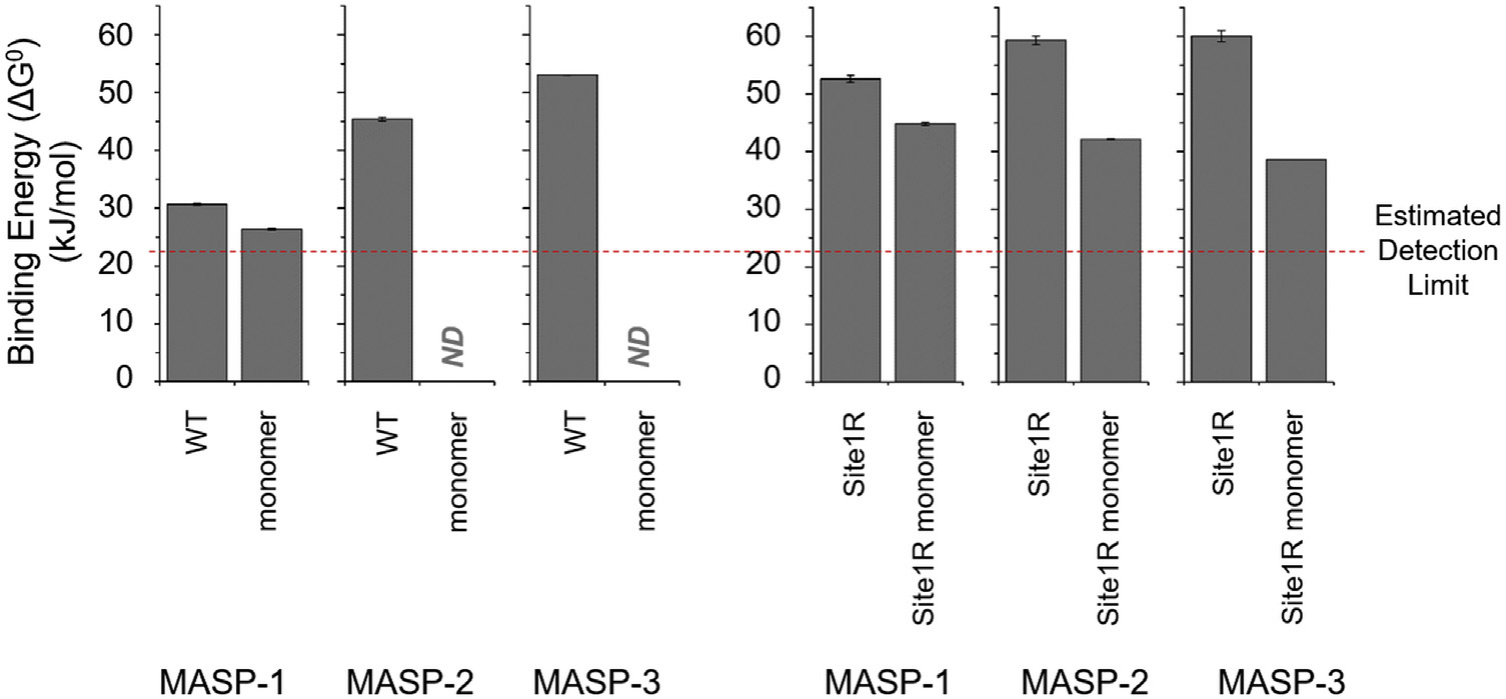
**The effect of the monomerization shows opposite site1-context dependence for MASP-1 *versus* MASP-2 and MASP-3.** Ecotin monomerization disrupts WT preorganization of site1/site2. The observed drop in binding energy is enzyme dependent and site1 dependent. For MASP-1, the drop is small and increases upon the site1R (P1 Met to Arg) mutation. For MASP-2 and MASP-3, the drop is large but decreases upon the site1R mutation. Results are the average of at least two measurements. The SD is shown by the error bars. MASP, mannan-binding lectin-associated serine protease.

As illustrated in Figure 9, the extent of binding energy loss upon abolishing site1/site2 preorganization is not only enzyme dependent but also site1 dependent. For MASP-1, the drop is small, 4.3 kJ/mol with P1 Met and 7.8 kJ/mol with P1 Arg; therefore, it increases with 3.5 kJ/mol upon the Met84Arg P1 change (data in Table 5). For MASP-2 and MASP-3, the drop is large and actually decreases upon Met84Arg P1 change. For the WT P1 Met, the drop is unmeasurable, but based on our 24.5 kJ/mol detection limit, it is at least 20.9 kJ/mol for MASP-2 and at least 28.6 kJ/mol for MASP-3. For the site1R, these values are 17.2 kJ/mol for MASP-2, which is at least 3.7 kJ/mol smaller, and 21.4 kJ/mol for MASP-3, which is at least 7.2 kJ/mol smaller (Tables 1 and 5, Fig. 9).

This interesting nonadditivity phenomenon further corroborates our model proposed for MASP-dependent WT ecotin-binding mechanism. MASP-2 and MASP-3 need to go through a slow conformational change to bind the P1 Met. However, the P1 Met84Arg replacement can change the association mechanism because of the newly emerged electrostatic complementarity between the positively charged P1 Arg and the negatively charged Asp (Asp189c) in the S1 pocket. As site1 association becomes faster and stronger, site1/site2 preorganization becomes less important, because the quickly formed heterodimers can readily form heterotetramers. This is also congruent with our size-exclusion chromatography results (Fig. 8), where the site1R monomer exclusively forms stable heterotetramers with the MASP enzymes with no signs of dimers or trimers. On the other hand, for MASP-1, P1 Arg does not lessen the contribution of site1/site2 preorganization, as site1 association does not require large conformational changes, it is already fast with P1 Met, and the S1 pocket in its self-inhibited state is not negatively charged because of an intramolecular salt bridge.

### Functional relevance of the observed phenomena: complement LP inhibitory capacity of the ecotin variants

**Figure 10:**
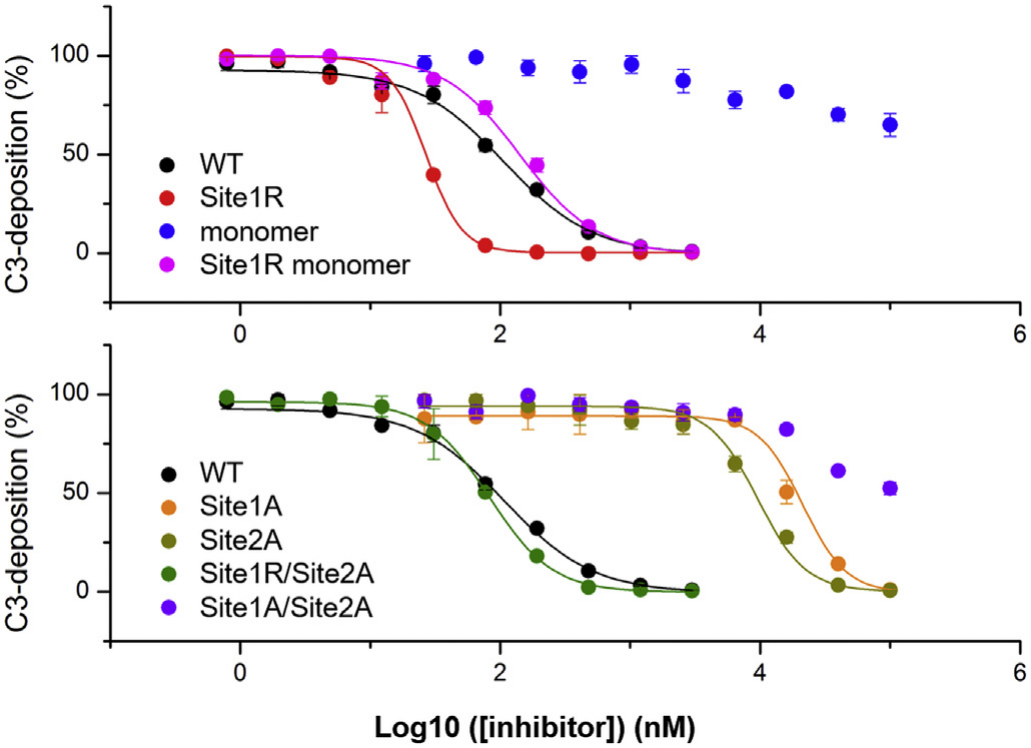
**Effects of mutations on complement lectin pathway inhibitory potency of ecotin.** The site1R variant containing the primary site-enhancing P1 M84R replacement has improved inhibitory potency. The same P1 M84R change in site1R/site2A can largely compensate for secondary site weakening or for erasing the preorganized binding sites in the site1R monomer. On the other hand, weakening the primary site or the secondary site either alone in site1A or in site2A mutants, respectively, or in combination in the site1A/site2A mutant, or erasing preorganization of site1 and site2 in the context of monomeric P1 Met ecotin render these variants practically ineffective against LP activation. *Symbols* represent the average of at least two measurements. Error bars represent the SD.

Complement LP activation depends on the activity of MASP-1 and MASP-2, and both enzymes are essential (17). Therefore, complete blocking of any of these enzymes halts LP activation. LP inhibitory potency of the eight tested ecotin variants in an LP-specific ELISA is illustrated in Figure 10, whereas the corresponding IC_50_ values are listed in Table 6.

**Table 6:** Complement LP inhibitory IC_50_ values of ecotin variants

Ecotin variant	IC_50_ (nM ± SE)		IC_50_ (nM ± SE)
WT	107.2 ± 1.1	Site1A	21,186.6 ± 1.1
Site1R	26.9 ± 1.0	Site2A	9543.9 ± 1.1
Monomer	ND	Site1R/site2A	83.2 ± 1.0
Site1R monomer	141.3 ± 1.1	Site1A/site2A	ND

Abbreviation: ND, inhibition is weak, thus the IC_50_ value could not be determined.

Of the seven ecotin mutants, two exceeded and one approached the efficacy of the WT inhibitor in blocking the LP. Importantly, all three had a P1 Arg. The most potent is site1R, which has an enhanced P1 combined with WT site2 in a stable dimeric structure. This mutant is followed by site1R/site2A, where site1 is boosted while site2 is weakened. Finally, the monomeric site1R having an affinity enhanced site1 but lacking preorganized binding site pairs is nearly as efficient as WT.

In all, an improved site1 can significantly boost LP inhibition for a dimeric ecotin with intact site2 and compensate for the lower site2 contribution in the case of the site2-weakened and the monomeric ecotin variants. All other forms having WT P1 (Met) and perturbed (either by site2A mutation or monomerization) site2 or a P1 Ala are either marginally effective or ineffective as LP inhibitors. This clearly shows that P1 Met of WT ecotin is inherently suboptimal for LP inhibition; therefore, the observed contribution of site2 and site1/site2 preorganization is essential for this ecotin function.

As the commercial Wieslab ELISA (31) can be viewed as a reference tool to test the efficacy of complement inhibitors, we measured a subset of the ecotin variants by WiELISA as well (Fig. S6). Despite that our in-house test and WiELISA use different serum samples and detect different complement activation products, the results are comparable.

### LP inhibitory potency of ecotin in 50% serum

**Figure 11:**
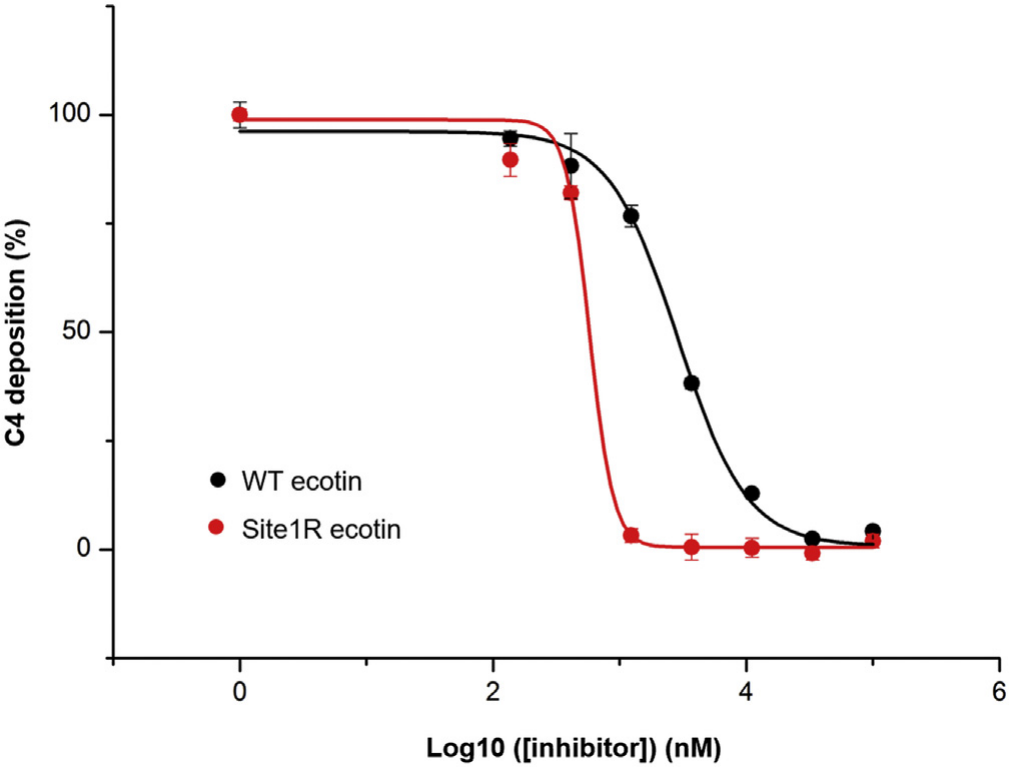
**LP-inhibitory potency of WT and site1R ecotin in 50% serum.** WT ecotin is able to completely block LP activation even in twofold diluted serum. The site1R mutation significantly increases the LP-inhibitory potency of ecotin. LP, lectin pathway.

As we have shown previously (16) and also in this article (Fig. 10), *E. coli* ecotin is a potent inhibitor of the LP in commonly used 2% human serum. Nevertheless, we also tested whether ecotin and its affinity-improved site1R variant can block the LP at more physiologic complement protein concentrations as well. We found that both inhibitors can inhibit the LP even in 50% serum (Fig. 11 and Table 7). As expected, the site1R variant has a significantly increased inhibitory potential.

**Table 7:** LP-inhibitory potency (in terms of IC_50_ values) of WT and site1R ecotin in 50% serum

Ecotin variant	IC_50_ (nM ± SE)
WT ecotin	2885.9 ± 1.5
Site1R ecotin	577.6 ± 1.1

While ecotin is able to completely block LP activation in twofold diluted serum, it requires inhibitor concentrations in the 10 μM range. This is, however, not only the result of the higher complement protein concentration. Both WT and site1R ecotin inhibit many serum proteinases unrelated to LP activation (9, 32), which decreases their “effective LP-inhibitory concentration.” While the site1R mutation boosts MASP-1 and MASP-2 inhibitory potency, it should also increase the binding affinity of ecotin to the competing “off-target” serum proteinases as well.

### Evolutionary aspects of the binding mechanism and target proteases of ecotin

Ecotin is capable of inhibiting the LP, mainly through the inhibition of MASP-2. However, a site1R mutant ecotin is a more potent LP inhibitor, which raises several questions. First of all, one might wonder, why do most ecotin orthologs have a P1 Met? The most plausible explanation is that ecotin-producing microbes need to be protected not only against trypsin-like but also against chymotrypsin-like and elastase-like host enzymes, including leukocyte elastase, and several pancreatic proteases (1, 10). A P1 Arg would compromise these latter functions.

Inhibiting MASP-1 with a more promiscuous “non-Arg” P1 is inefficient. MASP-1 is the professional activator of MASP-2 *in vivo*, and MASP-2 is the only member of the LP that can cleave C4. Consequently, LP activation can be controlled through inhibiting any one of these enzymes (17). However, deposition of the main opsonin C3 and formation of the terminal complement complex can be forestalled *via* the inhibition of MASP-2 without complete inhibition of MASP-1. These considerations might explain why natural evolution was biased for targeting MASP-2 instead of MASP-1, which demands a P1 Arg to be potently inhibited.

At higher serum concentration (Table 7) of ecotin inhibits *in vitro* LP activation at concentrations that might not be achieved *in vivo*. Nevertheless, it does not contradict its reported virulence factor role in pathogenic microbes including *E. coli* (10, 16, 33, 34) for several reasons. One is that the first encounter between host and pathogen usually takes place outside the circulation, such as on mucosal surfaces, where the concentrations of the complement components can be lower than in the plasma (35, 36, 37). Moreover, particular microbial mechanisms such as biofilm formation could lead to elevated local ecotin concentration in such microenvironments (38, 39). And finally, ecotin is only one of the many defensive tools of pathogens, and as such, ecotin by its own is not expected to shut down the LP activation in the blood.

The case of MASP-3 is peculiar both from mechanistic and evolutionary aspects. For long, this enzyme was recognized to be “inhibitor resistant” as neither canonical nor serpin-type substrate–like MASP-3 inhibitors were identified (40). This implied that the substrate-binding groove of MASP-3 is inaccessible for such inhibitors. The mere fact that evolution provided ecotin with high inhibitory efficacy against this challenging target enzyme suggests that MASP-3 inhibition provides great advantage for the ecotin-producing microbe. MASP-3 is the exclusive pro-FD activator in the blood, and this activity is essential for maintaining a functional AP (18). In fact, this is the only well-established immunological function of MASP-3. As complete and prolonged inhibition of MASP-3 in the circulation is an unlikely scenario by any pathogens, it implies that pro-FD–activating function of MASP-3 might be relevant in the extravascular environments as well, and its inhibition could be beneficial for the local ecotin-producing microbiota.

In all, ecotin-producing microbes inhibiting the LP and at least locally controlling the AP of many different hosts have a great adaptive advantage. *E. coli* is a widespread commensalist, and as MASP-2 and MASP-3 are evolutionarily more stable than MASP-1 (41, 42, 43), these are better targets for ecotin. We have already shown that ecotin also inhibits the LP of mice and rat (16), suggesting that inhibition of complement in the most different host organisms is a necessity for the microbes.

While MASP-1 is not a central target, low-efficient inhibitory potency of ecotin on MASP-1 might still be beneficial for the microbe in mammalian hosts as MASP-1 has several immunomodulatory, coagulation, and fibrinolytic functions unrelated to the LP (44, 45). Moreover, we have previously demonstrated that a broader specificity compound inhibiting both MASP-1 and MASP-2 (SFMI-1) can be a more efficient LP inhibitor than one being monospecific to MASP-2 (SFMI-2) (27). Therefore, natural evolution might have selected for a residual MASP-1 inhibitory potency of ecotin along the adaptive advantage of its crossspecificity and pan-specificity.

Natural evolution compensated for the suboptimal P1 Met by selecting a site2 region that targets mostly main chain moieties and binds to many different enzymes without large-scale conformational adjustments. Our results demonstrate that site2 provides essential contribution to MASP-2 and MASP-3 inhibition. This further corroborates that MASP-2 and MASP-3 are evolutionarily ancient and functionally important ecotin targets.

## Conclusions

**Figure 12:**
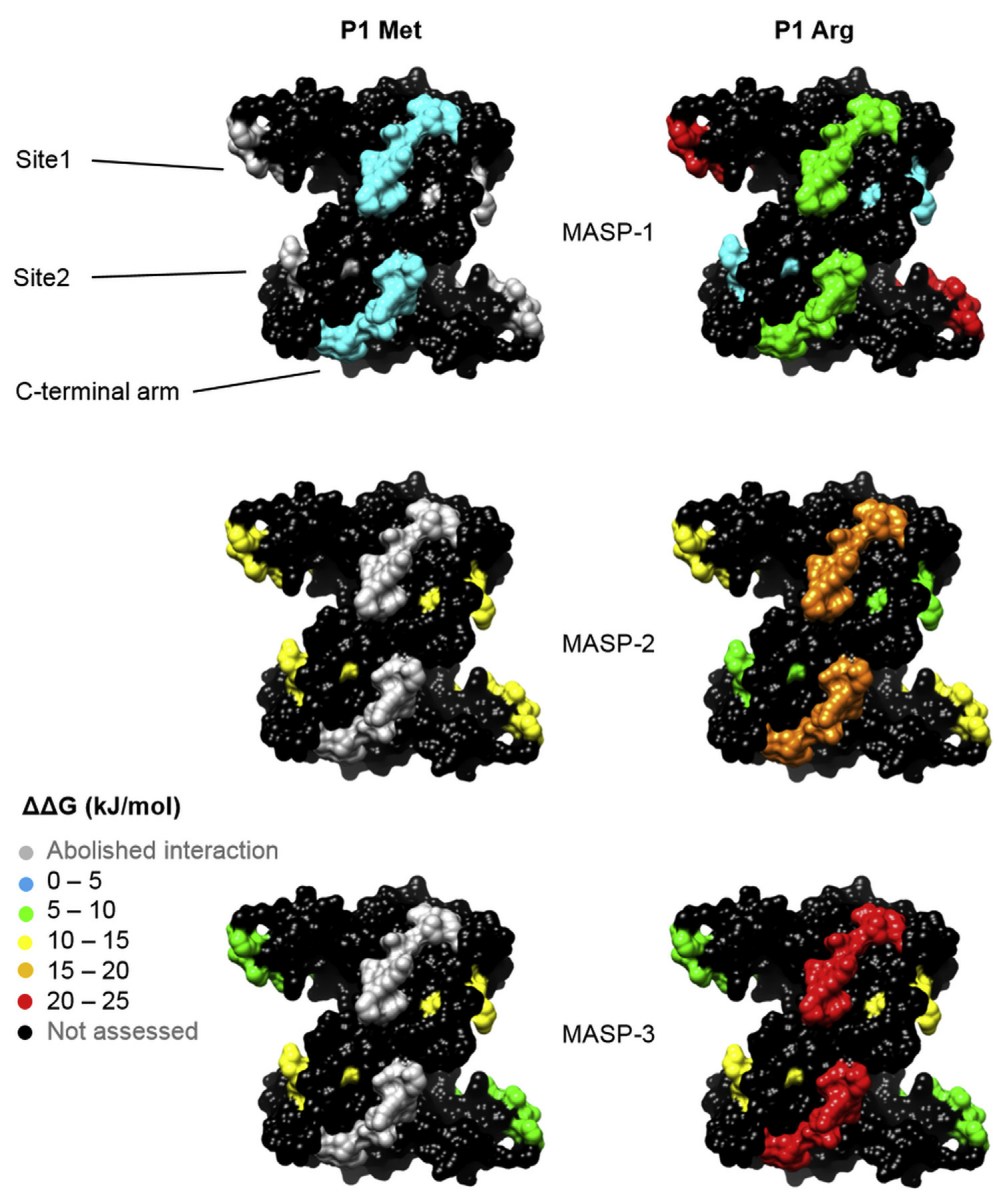
**Quantitative contribution of site1, site2, and site1/site2 preorganization to MASP inhibition mapped on the structure of ecotin.** For all three MASP enzymes, colors of site1, site2, and the C-terminal arms responsible for site1/site2 preorganization through stabilizing the ecotin dimer illustrate the extent of free energy change (ΔΔG) because of targeted alterations of these sites of ecotin in the context of P1 Met on the *left* and in the context of P1 Arg on the *right*. In the latter case, site1 is colored based on the effect measured upon replacing a P1 Arg with a P1 Met. The data are from Tables 2, 3, and 5. Color coding is explained on the *left*. *Gray* areas represent changes that abolish the interaction; nonassessed regions are colored *black*. P1 Arg elevates stability of all complexes and renders all binding site contributions measurable. In the P1 Arg context, site1 *versus* site2 and site1/site2 preorganization importance follow opposite trends for the enzymes: MASP-1 > MASP-2 > MASP-3 for site1 and MASP-3 > MASP-2 > MASP-1 for site2 and site1/site2 preorganization. Instead of showing ecotin in three slightly different conformations from the three MASP complexes, the figure was created based on the PDB ID 1EZU ecotin structure (5). MASP, mannan-binding lectin-associated serine protease; PDB, Protein Data Bank.

Our results generated new insights on the molecular mechanisms and evolutionary aspects of ecotin and have also shed light on how the substrate-binding machinery of the target MASP enzymes function. We applied “heat maps” (Fig. 12) for comprehensive yet compact visualization of MASP-dependent relative importance of the various ecotin sites.

The findings might also provide new therapeutic potentials as well. Because of the many biologic functions of the three MASP enzymes played in the complement system, the blood coagulation system, the fibrinolytic system, and the kininogen–kallikrein system as well as in endothelial activation (17, 44, 46, 47), ecotin emerges as an important element of the immune response–hindering arsenal of microbes. In fact, ecotin is a known virulence factor for several serious pathogens (33, 34, 48, 49). Note, that the majority of ESKAPE group pathogens causing the most frequent multidrug resistant infections express ecotin (50). Therefore, blocking the function of ecotin could deprive many pathogens from a key evolutionarily conserved immune-evading microbial mechanism. From this aspect, our most important finding is that MASP-2 and MASP-3 can be inhibited by ecotin only if site1 and site2 are preorganized in homodimer ecotin. In this regard, we demonstrated that monomeric ecotin, which is inactive on MASP-2 and hardly inhibits MASP-1, is unable to block the LP. Besides the obvious strategy to block ecotin site1 or site2, this finding provides new opportunity to combat dangerous multidrug-resistant pathogens through developing compounds capable of blocking ecotin dimer formation. Such compounds would render ecotin inactive against MASP-2, MASP-3, and most probably against many other ecotin targets and could be promising lead molecules of novel drugs.

## Experimental procedures

### Ethics statement

All studies using human serum were conducted according to the WMA Declaration of Helsinki. Experimental protocols were approved by the local ethics committee (permission number: TUKEB 9190-1/2017/EKU). Informed written consent was obtained for the isolation of peripheral venous blood from the donors.

### Bacterial strains and chemicals

Common reagents and constituents were from Bachem, New England Biolabs, Molar Chemicals Ltd, Sigma–Aldrich, and Thermo Fisher Scientific. The *E. coli* BL21 (DE3) Star cells were purchased from Invitrogen. Ecotin-KO line of *E. coli* BL21 (DE3) Star was prepared as described previously (16).

### Subcloning of the ecotin genes

WT or site1R ecotin genes in *pT7.7* vectors (11) were used as templates for PCR. Both genes were amplified by PCR using the following pair of oligonucleotides: 5′-CGAAATTAATACGACTCACTATAGGG-3′ and 5′-AATAGGGGATCCTTATTAGCGAACTACCGCG-3’. The PCR products were digested with NdeI and BamHI and ligated into the *pET25*b(+) phagemid vector. The resulting plasmids were sequenced. These DNA constructs allowed similar periplasmic expression to pT7.7, but in addition, they also enabled Kunkel-type site-directed mutagenesis.

### Site-directed mutagenesis

Single-stranded DNA form of the *pET25*b(+) vectors carrying the ecotin genes was isolated from *E. coli* CJ236 (New England Biolabs), and Kunkel mutagenesis was performed as described (51). Met84Ala replacement was introduced by the primer: 5′-GCCATCCGGGCAGGCCAT**AGC**CGTTGAAAC CGGGG-3′, whereas simultaneous Arg108Ala and Asn110Ala replacement was achieved with the primer 5′-CCACGATCGGCAGCTTGCT**CGC**GTA**GGC**CAGCATTCCAGC ATCGCCC-3’.

Bold indicates mutation sites. All constructs were sequenced before further use.

### Recombinant complement proteases

Catalytic fragments of MASP-1, MASP-2, and MASP-3 were expressed and purified as described previously (25, 40, 52).

### Expression and purification of monomeric ecotin variants

The monomeric forms of WT and site1R ecotin were expressed using the *pT7*.*7*-based vector construct described elsewhere (13). Freshly transformed *E. coli* BL21 (DE3) Star (Ecotin-KO) cells were grown in LB broth supplemented with 100 μg/ml ampicillin with vigorous shaking at 37 °C until reaching an absorbance of 4.0 at nm. Protein expression was induced by adding IPTG to a final concentration of 0.5 mM, and the cells were grown for 4 h at 37 °C. Then, the cells were chilled on ice and collected *via* centrifugation (4500*g*, 4 °C, 10 min), resuspended in 1/20 original culture volume ice-cold 1 mM MgCl_2_, and were frozen at −20 °C for at least 8 h. The cells were slowly thawed at 4 °C, cell debris was sedimented *via* centrifugation (48,000*g*, 4 °C, 20 min), and the supernatant representing the periplasmic fraction was collected. About 65 m/v% ammonium sulfate was added gradually to the supernatants, and the precipitated proteins were collected *via* centrifugation (48,000*g*, 4 °C, 20 min). The pellet was resuspended in 2.5 mM HCl and dialyzed against the same solution for 12 h at 4 °C. At this step, most contaminating proteins became precipitated and were removed *via* centrifugation (48,000*g*, room temperature [RT], 10 min). The pH of the supernatant was adjusted to 8.5 by adding Tris(hydroxymethyl)aminomethane (*Tris* base), and the samples were further purified on a 5 ml *HiTrap Q HP* anion-exchange column equilibrated with 20 mM Tris–HCl, pH 8.5 buffer (buffer A), on an ÄKTA Pure system at RT. The ecotin variants were eluted with increasing concentration of buffer B (20 mM Tris–HCl, pH 8.5, 1 M NaCl). The fractions containing the ecotin variants were identified by SDS-PAGE and were further purified on a *Jupiter 10u C4 300A* reverse phase chromatography column on a *Hewlett Packard Agilent Series 1100* device in water/acetonitrile solution system containing 0.1% trifluoracetic acid, with an increasing concentration of acetonitrile. The volatile components were eliminated by two rounds of lyophilization. Lyophilized ecotin was resuspended in water, the concentration of the solution was determined by measuring the absorbance at 280 nM in a Beckman Coulter DU-7400 photometer, and the sample was stored at 4 °C until use.

### Expression and purification of dimeric ecotin variants

The *pET25*b(+) expression constructs were transformed into *E. coli* BL21 (DE3) Star Ecotin-KO cells. Expression and purification of *E. coli* ecotin (UniProt ID: P23827) and its point mutants begun the same as for the monomeric ecotin forms, but after the gradual desalting with ammonium sulfate, the sample was dialyzed against the 2.5 mM HCl solution only for 3 h at RT. Precipitated contaminants were eliminated *via* centrifugation (48,000*g*, 4 °C, RT), and the supernatant was dialyzed against 20 mM Hepes, 150 mM NaCl, 5 mM CaCl_2_, 5 mM MgCl_2_, pH 7.5 (MASP kinetic assay buffer), overnight at 4 °C. The sample was centrifuged again (48,000*g*, 4 °C, 10 min), and ecotin was further purified *via* size-exclusion chromatography on a Superdex 200 Increase 10/300 GL column, on an ÄKTA Pure system, at RT, with a flow rate of 0.8 ml/min. The ecotin-containing fraction was dialyzed against 30 mM ammonium acetate at 4 °C overnight, and the dialyzed sample was lyophilized. Lyophilized ecotin was dissolved in water. Further treatment of the samples was as described for monomeric ecotin variants.

### Measuring the equilibrium inhibitory constants

Equilibrium inhibitory constants (*K*_I_) of the ecotin variants against the three MASP enzymes were determined by the method of Green and Work (53), modified by Empie and Laskowski (54), as described in detail previously (16). Kinetic measurements were carried out in MASP kinetic buffer supplemented with 0.1% Triton X-100 detergent. Cleavage of the substrate Z-L-Lys-Sbzl or Z-Gly-Arg-Sbzl was followed using 5,5′-dithiobis(2-nitrobenzoic acid) or Thiol Fluorescent Probe IV (Calbiochem) as cosubstrate on a BioTek Synergy H4 hybrid multimode microplate reader. Measurements were done in independent duplicates. Data were analyzed using the Origin Pro 8 software (OriginLab Corporation) as described elsewhere (16). Correction of apparent *K*_I_ values (*K*_I_∗) was carried out as described by Eggers *et al*. (10): when the observed *K*_I_ value was higher than 2 nM, the *K*_I_ value was interpreted as an apparent one, and the genuine *K*_I_ value was calculated as described, using previously published *K*_*M*_ values (16). However, in spite of the correction, many *K*_I_ values are still considered to be apparent ones because of the complex kinetics caused by the dimeric nature of ecotin (10, 14).

Using the equilibrium binding constant (*K*_I_) values, standard free energy (referred to as binding free energy) values were calculated according to the equation ΔG^0^ = −RT ∗ ln *K*_I_. Differences of binding energies between variants were expressed as ΔΔG values. All experiments were conducted at *T* = 298 K.

### Upper limit of *K*_I_ determination

The highest ecotin concentration we applied in the kinetic assays was 5 × 10^−5^ M. Above this concentration, nonspecific binding would generate artifacts. When the residual enzyme activity remained over 50% at such ecotin concentrations, we did not extrapolate to estimate a *K*_I_ value. Instead, we recorded that the *K*_I_ value is higher than 5 × 10^−5^ M, which corresponds to a binding free energy lower than 24.5 kJ/mol.

### Crystallization and structure determination of MASP-1:site1R ecotin and MASP-2:ecotin complexes

The activated recombinant catalytic fragments of MASP-2 (containing CCP2–SP domains) and MASP-1 (containing CCP1–CCP2–SP domains) were expressed and purified for crystallization as described elsewhere (52, 55). The CCP2–SP fragment of MASP-2 (at a final concentration of 2.7 mg/ml) was mixed with *E. coli* ecotin in 1:1 M ratio (corresponding to protease-binding sites) to allow stoichiometric complex formation. The complex was then crystallized by the hanging drop method at 20 °C by mixing 1.5 μl of protein complex solution and 1.5 μl of reservoir solution (17% [w/v] PEG 3350 and 0.3 M LiCl). Glycerol was used as a cryoprotectant.

The MASP-1:site1R ecotin complex was also crystallized by the hanging drop method at 20 °C by mixing 1 μl of protein complex solution (MASP-1 CCP1–CCP2–SP fragment, final concentration of 3.6 mg/ml) with equimolar site1R ecotin and 1 μl of reservoir solution (3.7 M NaCl, 0.1 M Hepes, pH 7.0). Glycerol was used as a cryoprotectant.

Two datasets were collected using helical data collection from a single crystal of MASP-2:*E. coli* ecotin complex at ESRF beamline ID23-1 at 100 K. Data were processed by XDS and XSCALE (56) to a resolution of 2.4 Å (Table S1). The asymmetric unit contains one MASP-2:ecotin_2_:MASP-2 heterotetramer, with the CCP2–SP domains being in slightly different relative orientations.

One dataset was collected from a crystal of the MASP-1:site1R ecotin complex at ESRF beamline ID23-2 at 100 K. Data were processed by XDS and XSCALE (56), and different resolution limits were carefully checked during refinement. A 3.4 Å resolution limit was chosen for processing (Table S1). The asymmetric unit contains one complete MASP-1:site1R ecotin_2_:MASP-1 heterotetramer and the half of a second one for which the complex is generated by crystallographic twofold rotation. The domains of the three MASP-1 molecules of the structure are in the same relative orientations, which facilitated noncrystallographic symmetry averaging of the electron density maps of the whole of three individual MASP-1 molecules, and for the three individual ecotin molecules, separately.

The phase problem was solved by Molrep program (57) of the CCP4 package (58) for both structures. As search models, structures of ecotin (PDB ID: 1AZZ), and domains of MASPs (for MASP-2, the SP and CCP2 domains from PDB ID: 1Q3X; for MASP-1, the SP domain and the CCP1–CCP2 segment from PDB ID: 3GOV) were used. Manual model building and refinement was carried out using Coot (59) and Phenix (60) packages, respectively. Refinement included translation/libration/screw refinement (translation/libration/screw groups were generated for individual domains of MASPs and for ecotin molecules) and noncrystallographic symmetry restraints (based on similar torsion angle values of the molecules, automatically generated by the program). Individual atomic *B*-factors were refined for the MASP-2:ecotin complex, whereas they were introduced instead of grouped *B*-factors in later stages of refinement for MASP-1:site1R ecotin complex. The models were validated using MolProbity (61). Figures were generated in PyMOL (v0.99, DeLano Scientific, 2006). Data collection and refinement statistics are compiled in Table S1. The structures of MASP-2:ecotin and MASP-1:site1R ecotin complexes have been deposited in the PDB under PDB IDs 7PQN and 7PQO, respectively.

Molecular contacts were analyzed using the CCP4 package and Coot.

### Analysis of the quaternary structure of enzyme:inhibitor complexes

Size-exclusion chromatography experiments were carried out on a Superdex 200 Increase 10/300 GL column, on an ÄKTA Pure chromatography system, at RT, with 0.8 ml/min flow rate in MASP kinetic assay buffer. The experiment was optimized to separate the enzyme:inhibitor complexes from the individual components. MASP-2 was adsorbed to the gel matrix at low salt concentration. This effect could be suppressed by increasing the NaCl concentration to 500 mM, indicating that the nature of the adsorption was electrostatic. Therefore, all MASP-2-related experiments were conducted by using a MASP kinetic assay buffer complemented with 500 mM NaCl. The modified condition did not alter the activity of MASP-2 and inhibitory potency of the ecotin variants. The *K*_I_ values for the site1A variant in original *versus* modified buffer were 1.4 × 10^−6^ M *versus* 1.16 × 10^−6^ M, whereas the same for the site2A variant were 9.75 × 10^−7^ M *versus* 5.8 × 10^−7^ M. Applying the ionic strength, MASP-2 showed the same elution volume as MASP-1 and MASP-3 in MASP kinetic assay buffer. Elution volume of ecotin was not affected (Fig. S4, *C* and *D*).

For the experiments, enzyme and inhibitor concentrations were set to exceed the respective *K*_I_ values with at least two orders of magnitude to promote complex formation, and the enzyme: inhibitor mixtures were incubated for 3 h at RT before loading onto the column.

### Human serum samples

Normal peripheral blood specimens were obtained from 10 healthy individuals after they had provided written informed consent, and the sera were prepared and stored as previously described (16).

### LP-specific ELISAs in 2% serum

Pathway-specific ELISA tests for assessing efficacy of the ecotin variants were carried out as described previously (16, 27). Greiner high-binding plates were coated with 10 μg/ml mannan in 50 mM sodium carbonate (pH 9.6) overnight at 4 °C. The remaining protein binding surfaces were blocked with 5 mg/ml bovine serum albumin, diluted in 20 mM Tris–HCl, 150 mM NaCl (Tris-buffered saline, pH 7.5), for 1.5 h at 37 °C. The sera were diluted to a final concentration of 2% in MASP kinetic assay buffer containing 0.1% Tween-20 and different concentrations of inhibitors and incubated for 30 min at RT. The plates were washed with Tris-buffered saline supplemented with 5 mM CaCl_2_ and 0.1% Tween-20 (wash buffer). The sera were applied to the plates and incubated at 37 °C for 30 min. After extensive washing with wash buffer, first an antihuman C3c antibody (DakoCytomation; catalog no.: A0062), then a secondary horseradish peroxidase–conjugated antibody (Sigma; catalog no.: A1949) was applied to detect deposited C3 fragments. A solution of 1 mg/ml o-phenlyenediamine and 1 μg/ml H_2_O_2_ in 50 mM potassium citrate (pH 5.0) was used as chromogenic substrate. The signal generated by uninhibited sera was considered to be 100% activity (positive control), whereas that of produced by sera supplemented with 20 mM EDTA was considered to be 0% (negative control). Utilizing Hepes instead of veronal buffer does not alter the outcome of serum experiments (62).

We also conducted comparative tests with the commercial Wieslab kit (31), in which we followed the protocol provided by SVAR Life Science.

The results were evaluated using the Origin Pro 8 software. The *DoseResp* function of the *Pharmacology* built-in function set was fitted to the data as described previously (16).

### LP inhibition tests in 50% serum

Inhibitory potency of ecotin in 50% diluted serum was measured as described previously (17). The plates were coated and washed as described for the measurements in 2% serum. The serum was supplemented with 0.1 mg/ml sodium polyanethole sulphonate as described previously (63), to selectively block the AP and CP without inhibiting the LP (17). Complement activation was measured through the detection of deposited C4 fragments *via* a specific anti-C4 antibody (DakoCytomation; catalog no.: Q0369). Further handling and development of the plate was as described for the experiments in 2% serum.

## Data availability

The atomic coordinates and structure factors of the MASP-2:ecotin and MASP-1:M84R ecotin complexes have been deposited in the PDB (http://wwpdb.org/) with accession codes PDB ID: 7PQN and PDB ID: 7PQO, respectively. All other data are contained in the article.

## Supporting information

This article contains supporting information (17).

## Conflict of interest

The authors declare that they have no conflicts of interest with the contents of this article.
