# Supplementary material for: Synergy of protease-binding sites within the ecotin homodimer is crucial for inhibition of MASP enzymes and for blocking lectin pathway activation
Source: J Biol Chem. 2022 Apr 25;298(6):101985. doi: 10.1016/j.jbc.2022.101985 (PMC9136129; doi:10.1016/j.jbc.2022.101985)
Supplement: Nagy_et_al_Supp_Inf_REVISION-2-CLEAN-2022-04-19.Docx [file mmc1.docx]

**Synergy of protease binding sites within the ecotin homodimer is crucial for inhibition of MASP enzymes and for blocking lectin pathway activation**

**Supporting information**

| **Complex** | **MASP-2/ecotin** | **MASP-1/ Site1R ecotin** |
| --- | --- | --- |
| **Data collection** |  |  |
| Beamline,  wavelength (Å) | ESRF ID23-1,  0.97942 | ESRF ID23-2,  0.8726 |
| Unit cell parameters a,b,c (Å) | 99.562, 102.746, 109.938 | 251.066, 251.066, 211.585 |
| Space group | P2_1_2_1_2_1_ | I422 |
| Resolution (Å) * | 30.0-2.40 (2.50-2.40)* | 40.0-3.40(3.49-3.40) |
| No. of unique / observed refl. | 44699 /236730 (5067 /17854) | 45525 /341362 (3058 /16950) |
| < *I / σ(I)* > | 13.50 (2.36) | 13.02 (0.74) |
| *R*_meas_ | 0.094 (0.755) | 0.021 (2.55) |
| Completeness (%) | 99.7 (99.4) | 97.1(90.1). |
| CC(½) (%) | 99.8 (75.9) | 99.7(59.5) |
| **Refinement** |  |  |
| Resolution range (Å) | 29.8-2.40 | 39.6-3.39 |
| *R / R*_free_ (No. of obs.) | 0.1942 / 0.2329(44625/2217) | 0.2588/0.2969 (45287/2261) |
| No. of atoms: protein / ligand /  solvent | 6753 / 18 / 116 | 11330 / 43 / 0 |
| B-factor of MASP/ ecotin / ligands / solvent  (Å^2^) | 57.9 /58.4 / 70.9 / 48.7 | 137.4 / 126.7 / 153.2 / - |
| RMS dev. bond length (Å) | 0.007 | 0.002 |
| RMS dev. bond angles (°) | 0.883 | 0.55 |
| Ramachandran fav. / all. / disall. (%) | 97.42/ 2.47 / 0.11 | 95.23 / 4.71 / 0.06 |
| **PDB code** | **7PQN** | **7PQO** |

**Table S1. X-ray diffraction data collection and refinement statistics.**

*Data for the highest resolution shell is shown in parentheses.

**Table S2. Disordered and partially disordered residues in the MASP-1 : Site1R ecotin structure.** 2.8% of the amino acid residues are disordered (bold, underlined), for 22.2% of the residues the side chain is disordered (regular font; partially disordered side chain: in italics). Heterotetramer 1 is assembled from chains A:I:J:B; heterotetramer 2 is assembled from chains C:K and their symmetry equivalents.

| MASP-1 protein chains | C | A | B |
| --- | --- | --- | --- |
| Expression tag  CCP1 domain  (residues 298-365) | **294-297**  **298** 300 303 304 305 311 *312* 313 315 318 320 322 323 325 326 328 330 331 *333* 334 336 337 338 339 343 347 348 350 351 358 359 363 *364* 365 | **294-296** 297  300 303 304  311 *312* 313 315 316 318 320 322 326 328 331 334 335 336 337 338 339 340 343 346 *347* 350 351 *358 359* 363 *364* | **294-297**  **298-299** 300 **303-305** 308 309 311 312 313 **315-319** 320 322 323 325 326 328 330 *333* 334 335 336 *337* 338 339 340 341 343 346 347 348 350 351 357 358 361 363 364 365 |
| CCP2 domain  (residues 366-435) | 368 372 373 377 381 383 386 390 392 *393* 394 398 403 405 406 407 409 411 419 423 425 427 429 | 368 372 377 *378* 381 383 384 385 386 390 392 *393* 394 399 *403* 406 407 409 411 417 419 *423* 425 429 | 368 372 373 374 377 378 383 384 386 390 391 392 393 394 403 404 405 406 408 409 411 413 417 419 421 423 425 427 *429* 433 435 |
| SP domain  (residues 436-699) | 440 **441-448** 457 *494 499* 504 536 580 *585* 591 594 616 *622 623 624 627 630* 635 636 655 656 674 675 *677 688 698* | **444-448** 453 *456* 457 *472* 494 495 497 499 504 *515* 528 536 563 575 579 585 591 596 616 622 624 627 630 635 654 655 656 675 688 *693* 698 | 438 440 441 **442-448** 450 451 453 456 457 463 *469* 470 *472* 480 494 495 504 509 *520* 525 528 536 560 563 564 573 580 585 587 *591* 592 594 596 605 606 615 616 621 622 623 624 *627* 635 *636* 639 653 654 655 657 659 661 674 675 676 *677 684 688 691 693* 698 |
| Ecotin M84R protein chains  (residues 1-142) | K | I | J |
|  | **1-3** 9 *10* 18 *25* 31 32 47 58 *62* 76 91 92 94 *95* 103 108 112 *131*134 135 | **1-3** 4 5 9 *10* 18 25 31 37 47 58 *62* 76 91 *92* *93* 94 *95* 108 112 *115* 131 *133*134 135 137 *142* | **1-3** 4 5 *8* 9 18 21 *26* 31 *42* 47 58 60 *62* 65 76 78 79 91 *92* 93 94 95 103 112 131 *133* 134 135 |

**
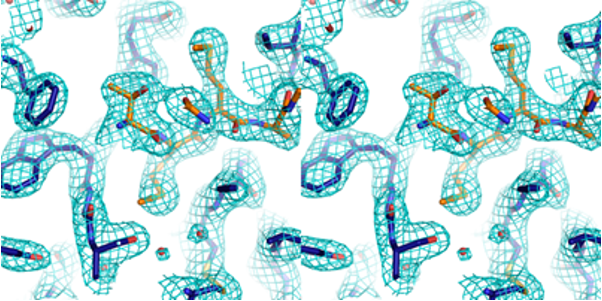
**

**Figure S1. 2*mF_o_*-*DF_c_* type electron-density map of the crystal structure of MASP-2:ecotin complex (PDB id: 7PQN) contoured at 1.5σ level (stereo view).**The electron density map is shown at the S1 site of the enzyme (MASP-2: dark blue, ecotin: orange).

**
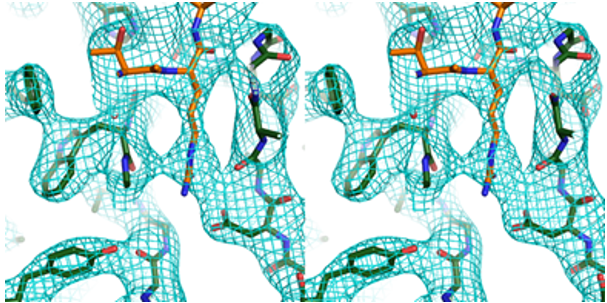
**

**Figure S2. 2*mF_o_*-*DF_c_* type e electron-density map of the crystal structure of MASP-1:Site1R ecotin complex (PDB id: 7PQO) contoured at 1.5σ level (stereo view).** The electron density map is shown at the S1 site of the enzyme (MASP-1: dark green, Site1R ecotin: orange).

**
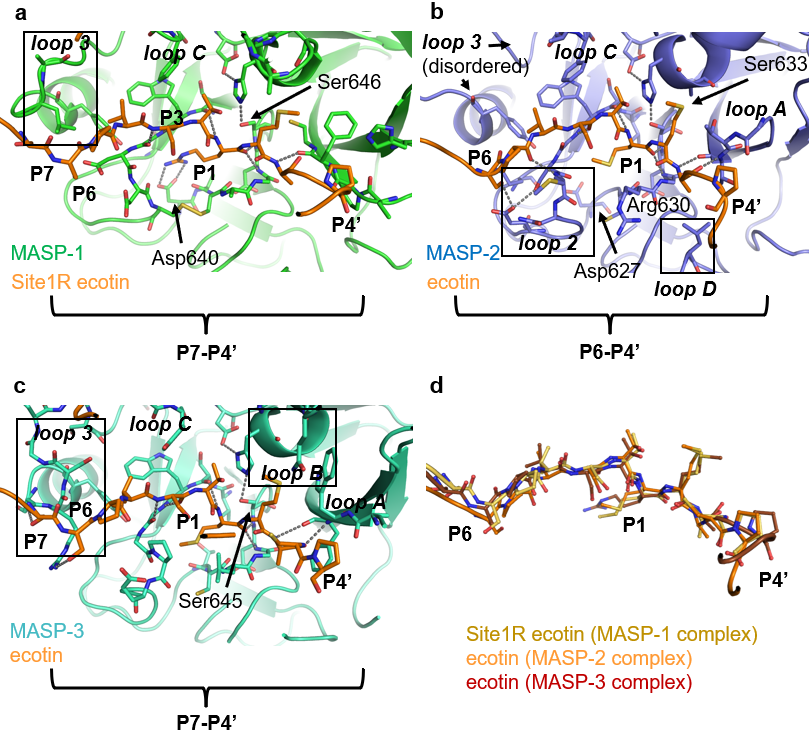
**

**Figure S3. The canonical arrangement of the 80s loop of ecotin within three MASP : ecotin complexes.** All three complexes show extensive interactions between the enzyme and the canonical loop of the inhibitor. At the core P4-P1 segment, the same backbone H-bonds exist with all three MASPs. In the MASP-1 : Site1R (M84R) ecotin complex, the intramolecular self-inhibitory salt bridge of MASP-1 is broken, as Asp640 (Asp c189 by chymotrypsinogen numbering) of the enzyme forms an intermolecular salt bridge with the P1 Arg of the inhibitor (a). In the MASP-2 : ecotin (b) and MASP-3 : ecotin (c) complexes, the P1 Met does not fully occupy the S1 pocket of the enzyme. For MASP-2, the interacting segment of the 80s loop of ecotin is somewhat shorter than for MASP-1 and -3, and furthermore, a significant structural rearrangement occurs upon complex formation with ecotin resulting in disorder of loop3 (b). The tight overlap of the canonical ecotin loops in the three MASP : ecotin complexes illustrates that the backbone conformation of the 80s loop is highly similar in spite of the different accommodation at the ends of this loop in these complexes(d).

**
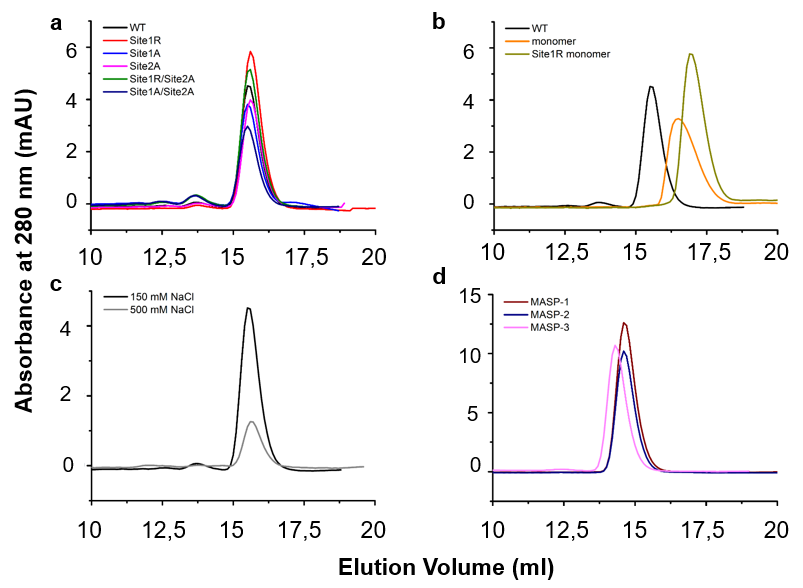
**

**Figure S4. Point mutations do not alter the quaternary structure of ecotin.** Site1 and Site2 mutant ecotin variants show the same elution volume in size exclusion analysis, suggesting that the corresponding point mutations do not affect the wild type homodimeric structure of ecotin (a). These variants have a significantly smaller elution volume than the monomeric forms (b). For the analysis of MASP-2 complexes, we had to use a buffer supplemented with 500 mM NaCl to suppress a weak electrostatic interaction between MASP-2 and the gel matrix. Increasing the ionic strength did not alter elution volume of ecotin. Please note that difference between peak sizes is due to the different amount of ecotin used in these tests (c). The MASP-1 and -3 enzymes were analyzed in MASP kinetic buffer, while for MASP-2 it was supplemented with 500 mM NaCl. In these conditions, the three MASPs showed practically the same elution volume (d).


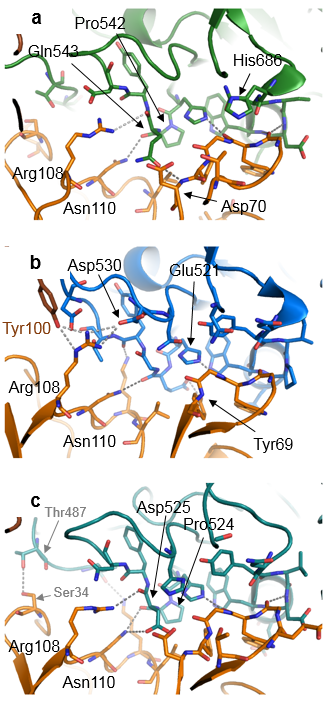


**Figure S5. Overall look of the contacts formed between ecotin Site2 and the three MASPs.** Both the 60s and 100s loops of the Site2 of ecotin (orange) are in contact with the MASPs (MASP-1: green, MASP-2: blue, MASP-3: turquoise). However, there is a more extended network of contacts in the MASP-2 : ecotin (b) and MASP-3 : ecotin (c) complexes compared to that in the MASP-1 : Site1R ecotin complex (a). An exceptional feature of the MASP-2 : ecotin complex is that ecotin Arg108 contacts not only the sidechain of MASP-2 Asp530 but also Tyr 100 (highlighted with colored label) of the other ecotin protomer.

**Table S3. List of contacts formed between the MASP enzymes and ecotin Site2.** Residues within 4.0Å distance of ecotin 60s loop and 100s loop are listed, with residues forming hydrogen bond (donor and acceptor atoms within 3.5Å distance) shown in bold. PDB structures used: MASP-1 : Site1R ecotin: 7PQO; MASP-2 : ecotin: 7PQN; MASP-3 : ecotin: 4IW4. Non-crystallographic symmetry related molecule pairs are shown in different columns.

|  | | **MASP-1 / Site1R ecotin  contacts** | | | **MASP-2 / ecotin contacts** | | **MASP-3 / ecotin contacts** | |
| --- | --- | --- | --- | --- | --- | --- | --- | --- |
| Contacting chains | | J..A | I…B | K…C* | D..A | C..B | C..F | D..E |
| No. of contacts | | 63 | 53 | 66 | 69 | 59 | 88 | 89 |
| H-bonded resid. pairs | | 6 | 6 | 6 | 8 | 7 | 12 | 10 |
| ***Ecotin residue*** | | ***MASP-1 residue*** | | | ***MASP-2 residue*** | | ***MASP-3 residue*** | |
|  | 9 |  |  | **693** |  |  |  |  |
|  | 10 |  |  | 693 |  |  |  |  |
|  | 32 | 504 |  |  |  |  |  |  |
|  | 34 | 502 |  | 502 |  |  | **487** | **487** |
| 60s loop | 63 | 686 | 686 | 686 |  |  | 626 | **626** |
|  | 65 |  |  |  |  |  | 558 **690** |  |
|  | 66 | 686 687 688 **689 690** | 686 687 688 **689 690** | 686 687 688 689 **690** | 674 675 676 677 **678** | 674 675 676 677 **678** | 687 688 689 **690 691** | 687 688 689 **690 691** |
|  | 67 | **541** 686 *687* **689** 693 | **541** 686 *687* **689** 690 693 | **541** **686** *687* **689** 690 693 | **520** 674 677 681 | **520** 674 675 677 681 | **523** 687 688 **690** 691 694 | **523** 687 688 690 691 694 |
|  | 68 | 686 687 | 686 687 | 543 686 687 | 615 520 531 | 520 531 | 523 525 533 687 688 | 523 525 533 687 688 |
|  | 69 | 690 | 543 690 | 542 **690** | 520 **521** 678 | 520 **521** 678 | 523 524 525 691 | 523 524 691 |
|  | 70 | **543** | **543** | 543 |  |  | **525 533** | **525** |
| 100s loop | 108 | 545 |  |  | **526 530** | 526 (**100****) | **525** 526 527 | 526 **527** |
|  | 110 | **542** | **542** | **542** | **521 524** | **521** | **524 525** | **524 525** |
|  | 112 |  |  |  | 520 521 **523** | **520** 521 **523** | **489** 524 | **489** 524 |
|  | 113 | 542 543 | 542 543 | 542 543 | 521 522 | 521 522 | 524 525 | 524 |

* Crystallographic symmetry equivalent molecule

** Other MASP-2 in the heterotetramer


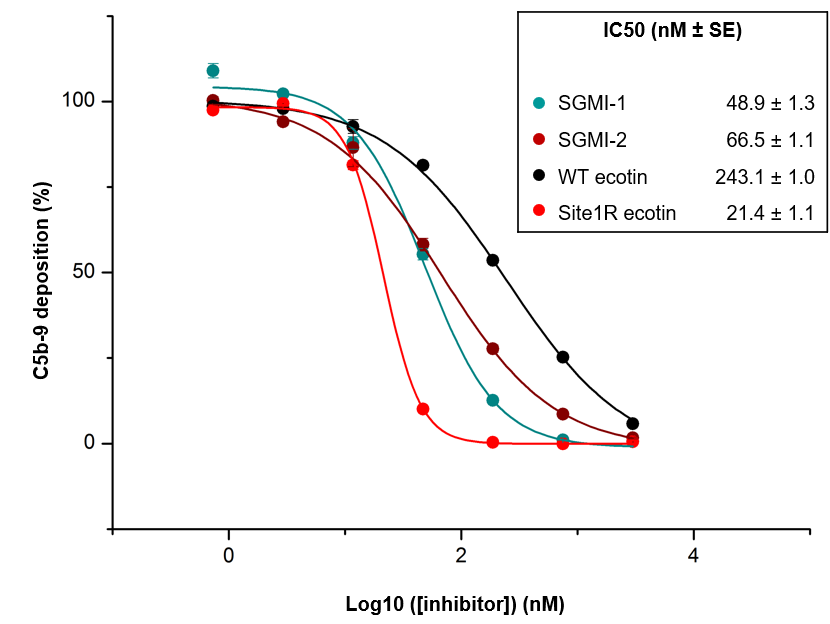


**Figure S6. WT and Site1R ecotin inhibit the LP in WiELISA assay.** The Wieslab ELISA assay (WiELISA) is a commercial test often used to assess complement deficiencies or the potency of complement inhibitors. Compared to our ‘in house’ assay, the WiELISA test applies different serum sample and detects different complement activation products. The fact that this test also finds WT and Site1R ecotin to be effective LP inhibitors and shows that the Site1R variant is more efficient, validates our ‘home-made’ assay results. We included our *in vitro* evolved SGMI-1 and SGMI-2 as internal controls that had been previously shown to potently inhibit the LP in WiELISA assays (https://doi.org/10.1073/pnas.1202588109).
